# Molecular Epidemiology and Genetic Characterization of Feline Panleukopenia Virus among Clinically Suspected Domestic Cats in Tamil Nadu, India

**DOI:** 10.3390/microorganisms14081642

**Published:** 2026-07-27

**Authors:** Raja Paramasivam, Ravi Vijayalakshmi, Mani Saminathan, Manoharan Parthiban, Vivekanandan Vinitha, Munisamy Chandrasekar, Viswanathan Naveenkumar, Aswathy Nair, Farnaz Nabiya, Swetha Saravanan, Dhananchezhiyan Sekar, Gopal Dhinakar Raj

**Affiliations:** 1 Department of Animal Biotechnology, Faculty of Basic Sciences, Madras Veterinary College, Tamil Nadu Veterinary and Animal Sciences University, Chennai 600007, Tamil Nadu, India; raviviji01@gmail.com (R.V.); drparthiban66@gmail.com (M.P.); vinithav26@yahoo.in (V.V.); nair99aswathy@gmail.com (A.N.); nabiyafarnaz@gmail.com (F.N.); swethas6300@gmail.com (S.S.); chezhiyanra18@gmail.com (D.S.); dirtrpvb@gmail.com (G.D.R.); 2 Division of Virology, ICAR-Indian Veterinary Research Institute, Mukteswar, Nainital 263138, Uttarakhand, India; 3 Department of Veterinary Medicine, Madras Veterinary College Campus, Tamil Nadu Veterinary and Animal Sciences University, Chennai 600007, Tamil Nadu, India; chandrasekar.m@tanuvas.ac.in; 4 Veterinary Clinical Complex, Veterinary College and Research Institute, Tamil Nadu Veterinary and Animal Sciences University, Udumalpet 642205, Tamil Nadu, India; naviviswanathan300@gmail.com

**Keywords:** feline panleukopenia, epidemiological factors, VP2 gene, phylogenetic analysis, molecular docking, domestic cats

## Abstract

Feline panleukopenia virus (FPV) causes feline panleukopenia (FP), a highly contagious and often fatal disease in domestic cats. This study investigated FPV detection, associated epidemiological factors, and partial VP2 genetic characteristics among clinically suspected cats presented in Chennai, Tamil Nadu, India. Exploratory molecular docking was also performed to compare predicted VP2–transferrin receptor type-1 interactions in feline and canine systems. FPV DNA was detected in 83 of 180 clinically suspected cats (46.11%). Multivariable analysis revealed higher odds of FPV PCR positivity in female cats than in male cats (aOR: 2.65; 95% CI: 1.35–5.21) and lower odds in vaccinated cats than in unvaccinated cats (aOR: 0.41; 95% CI: 0.17–0.97). Compared with summer, higher odds were observed during the southwest monsoon (aOR: 3.59; 95% CI: 1.26–10.20) and northeast monsoon (aOR: 17.11; 95% CI: 3.86–75.80), whereas age group and breed were not significantly associated with PCR positivity. FPV was successfully isolated from four of the ten PCR-positive clinical samples inoculated onto CRFK cells. Partial VP2 sequencing of 13 PCR-positive samples showed 98.1–98.5% nucleotide identity with the vaccine reference strain and identified several nucleotide and amino acid substitutions. Most study sequences clustered with Indian FPV reference strains, whereas one sequence clustered separately within the carnivore parvovirus grouping. Molecular docking of the (TfR1-VP2 interaction identified differences in the predicted interaction patterns between feline and canine complexes, with the feline complex exhibiting a more favourable ZRANK score. However, these findings represent computational predictions and require experimental validation. Overall, the study highlights the circulation of genetically diverse FPV strains in southern India and underscores the importance of continued surveillance and molecular characterization to support disease monitoring and control strategies.

## 1. Introduction

Cats are among the most important companion animals worldwide and are second only to dogs in popularity in developing countries such as India. Despite the availability of imported vaccines against feline panleukopenia (FP), information regarding the efficacy of these vaccines against circulating strains remain limited. Feline enteric viruses (FEVs) comprise a group of pathogens capable of causing gastrointestinal disorders in cats worldwide. The most important enteric viruses affecting cats include feline parvovirus or feline panleukopenia virus (FPV), feline coronavirus (FCoV), and feline calicivirus (FCV) [1]. Domestic, stray, and wild cat populations are more susceptible to these infections because of their endemic nature, seasonal occurrence, high mortality, and other epidemiological variables. Although vaccines against major feline viral diseases [FPV, FCV, and feline herpesvirus (FHV)] are commercially available, their effectiveness remains uncertain, as disease outbreaks continue to be reported globally, including among vaccinated animals.

Canine parvovirus (CPV) and FPV belong to the same taxonomic group [2]. After overcoming species barriers and accumulating five to six amino acid alterations in the capsid protein gene, CPV diverged from FPV in the late 1970s [3]. The original CPV-2 subsequently evolved into the currently circulating subtypes, CPV-2a- and CPV-2a-derived variants within a relatively short period. Although cats are generally resistant to the original CPV-2 strain, the newer CPV variants can infect felines and produce clinical signs that are indistinguishable from those caused by FPV infection [4,5].

Feline panleukopenia is a highly contagious and frequently fatal viral disease affecting both domestic and wild felines [6]. FPV possesses a single-stranded DNA genome of approximately 5kb containing two major open reading frames that encode the non-structural (NS1 and NS2) proteins and the capsid proteins (VP1 and VP2) [7]. Among the carnivore parvoviruses, FPV exhibits a broad host range and is associated with high pathogenicity, and a high rate of morbidity and mortality along with a quick clinical phase and dissemination [8]. The VP2 protein is the primary protein that contains significant epitopes, which stimulate the production of neutralizing antibodies. Further, the mutations in the VP2 gene can affect the antigenic properties and species tropism [9]. FPV exhibits a strong tropism for rapidly multiplying cells, particularly enteric epithelium, haemopoietic progenitor cells and cerebellar cells, resulting in clinical manifestations such as frequent emesis, melena, hematochezia, foul-smelling diarrhoea, and incoordination. The kittens born to infected pregnant queens commonly develop cerebellar hypoplasia [10]. FPV is transmitted primarily through the fecal-oral route and spreads through direct or indirect contact with infected body fluids, faeces, or other fomites as well as by fleas, mechanically. The virus is highly resistant in the environment and can survive at least up to one year in infected organic material [11].

Information regarding the prevalence and molecular epidemiology of FPV in India remains limited and geographically fragmented. The first report of FPV in Chennai, Tamil Nadu, identified the virus in fecal samples collected from clinically suspected cats between 2012 and 2013, with a prevalence of 25.37% (17/67) [12]. Subsequently, a study conducted in Kerala reported a prevalence of 77.78% (21/27) among clinically suspected cats using PCR-based [13]. Using feline fecal samples from 2011 to 2014, a cumulative incidence of 11.3% of FPV infections from different Indian states was reported [14]. A serological survey conducted in the Wayanad district of Kerala between 2017 and 2019 detected anti-FPV antibodies in 29.11% of unvaccinated healthy cats. More recently, a prevalence of 7.14% was reported among fecal samples collected from cats in Chennai between March and June 2022 [15].

Several molecular studies have highlighted the genetic diversity of FPV circulating in different geographical regions. Two characteristic amino acid variations in the VP2 gene of at positions 103 (valine) and 323 (aspartic acid) of the VP2 gene were reported in Bangladeshi FPV strains [16]. In another study, complete genome sequencing of an Indian FPV isolate demonstrated 99.75% nucleotide identity with a previously reported Indian variant of the virus [15]. The deduced amino acid sequence of the isolate revealed specific amino acid substitutions (Pro5Ala, Phe6Val, His7Gln, Asn9Asp, Lys16Arg, Lys19Arg, Asn52Lys, Gly58Trp, Thr66Ser, Lys67Arg, Leu70His, Asn373Asp, and Ala390Thr), when compared with other FPV strains. These findings suggest ongoing genetic diversification among circulating FPV strains and highlight the need for continued molecular surveillance to monitor viral evolution and vaccine-related genetic differences [15].

To date, systematic documentation of FPV from the FEV suspicion has not been reported in India, although veterinary hospitals across the country have documented several cases of feline gastrointestinal illnesses. Management of affected cats becomes more challenging when enteric viral infections are underdiagnosed or overlooked, despite the availability of numerous diagnostic methods. Early diagnosis and appropriate supportive therapy can improve survival in more than 90% of affected cats. Several diagnostic assays are available for the detection of FPV, including polymerase chain reaction (PCR), enzyme-linked immunosorbent assay (ELISA), electron microscopy, immunohistochemistry, and isothermal amplification techniques. These assays can be applied to a variety of clinical specimens, including in faeces, conjunctival and oral swabs, blood, ascitic fluid or tissues such as spleen and intestine, depending on the clinical presentation and infection stage [17,18,19].

Virus isolation remains a cornerstone technique for confirming active infection and obtaining viable viral stocks for further characterization of FPV. Although PCR-based assays enable rapid detection, recovery of infectious virus in cell culture is essential for studying viral pathogenicity, antigenic variation, and molecular evolution. The Crandell-Rees feline kidney (CRFK) cell line has been widely used for isolation and propagation of FPV due to its high susceptibility to feline, with characteristic cytopathic effects (CPE) such as cell rounding, shrinkage, and detachment observed within 48–72 h post-inoculation (hpi). Successful isolation of FPV from clinical and wild felid samples using CRFK cells has been documented, followed by confirmation through PCR amplification of the VP2 gene and sequencing analysis. Similarly, several recent investigations have demonstrated that field strains recovered in CRFK cells exhibited genetic variability in the VP2 region, highlighted the importance of continuous virus isolation for epidemiological surveillance and vaccine efficacy assessment [20]. Therefore, isolation of FPV in CRFK cells remains a gold standard approach for confirming infection and facilitating downstream molecular and biological characterization studies.

In addition to genetic characterization, understanding virus-host receptor interactions may provide insights into factors associated with host range and cross-species transmission. The transferrin receptor type-1 (TfR1) serves as the primary cellular receptor for FPV and related carnivore parvoviruses, with the VP2 capsid protein playing a key role in receptor recognition. Variations in VP2 have been associated with differences in receptor binding and host range among parvoviruses. Recent advances in computational structural biology, including AlphaFold-based structure prediction and molecular docking, have enabled in silico exploration of virus–receptor interactions. In the present study, molecular docking of TfR1 and VP2 was employed as an exploratory approach to compare the predicted interaction patterns between feline and canine systems and to identify residues potentially involved in receptor binding.

The present study aimed to investigate the prevalence, diagnostic sampling strategies, risk factors, genetic diversity, and nucleotide and amino acid variations of FPV strains circulating among clinically suspected cats in Chennai, Tamil Nadu, southern India, from September 2023 to September 2024. In addition, molecular docking analyses were performed to provide preliminary insights into the predicted interactions between VP2 and host TfR1 receptors. However, these computational findings should be intended as exploratory observations and require further experimental validation to establish their biological significance.

## 2. Materials and Methods

### 2.1. Study Settings

The study was conducted on clinically suspected feline panleukopenia cases presented to the Madras Veterinary College Teaching Hospital (MVCTH) and Private Pet Clinics, Chennai, Tamil Nadu, India from September 2023 to September 2024. The study population comprised cats presenting with clinical signs consistent with feline panleukopenia infection. The clinical suspicion criteria for FPV infection include vomiting, watery to haemorrhagic diarrhoea, dehydration, anorexia, depression, and fever [11,21]. These clinical suspicion criteria were evaluated by clinicians specialized in feline infectious diseases.

### 2.2. Sample Collection

A total of 180 clinically suspected cats were included during the study period, and various clinical samples (fecal, nasal, oral and conjunctival swabs, and blood) were collected from live animals. Details of the collected samples and the associated the risk factors are provided in Supplementary Table S1. All swab samples were resuspended in 1 mL of sterile phosphate buffer solution (1X PBS) and stored at −20 °C until nucleic acid extraction.

### 2.3. Necropsy Examination

Systematic necropsy examination were performed on deceased animals as per the standard protocol. Gross pathological lesions were recorded. Intestinal tissue samples were collected during necropsy, transported on ice, and immediately stored at −80 °C until PCR analysis.

### 2.4. Epidemiological Variables

Epidemiological variables evaluated for their association with FPV PCR positivity included sex, age group, breed, vaccination status, and season. Age was categorized as <3 months, 3–6 months, 6–12 months, 1–3 years, and >3 years. Breeds with small sample sizes were combined as “other breeds” for regression analysis. Seasons were classified as winter (January–February), summer (March–May), southwest monsoon (June–September), and northeast monsoon (October–December). Clinical signs, specimen types, and month-wise occurrence were summarized descriptively.

### 2.5. RNA Extraction and PCR Amplification

DNA and RNA extractions from the samples were done using a QIAamp DNA mini kit (QIAamp, Hilden, Germany) and QIAamp viral RNA mini kit (QIAamp, Hilden, Germany), respectively as per the manufacturer’s instructions. PCR amplification was performed using FPV-specific primers targeting the VP2 gene for the detection of FPV. The primers used were FPV-F (5′-GCTTTAGATGATACTCATGT-3′) and FPV-R (5′-GTAGCTTCAGTAATATAGTC-3′) targeting VP2 gene [16] with the product size of 698 bp. PCR amplification was performed with the following cycle conditions: initial denaturation at 94 °C for 4 min; 35 cycles of denaturation at 94 °C for 30 s; annealing at 55 °C for 30 s; extension at 72 °C for 1 min; and final extension at 72 °C for 7 min. Further to rule out possibility of mixed infections of other feline viral diseases, all the FPV-positive samples were subjected to PCR for detection of feline herpesvirus, feline coronavirus, and feline leukemia virus. 

### 2.6. Virus Isolation in CRFK Cell Line

For virus isolation, ten PCR-positive clinical samples (fecal swabs and intestinal tissue homogenates) with adequate sample volume and appropriate preservation quality were selected based on clinical signs and severity of the infection as suggested by the clinician and samples were processed under aseptic conditions. Approximately 10% (*w*/*v*) suspensions were prepared in sterile PBS (pH 7.2) containing antibiotic–antimycotic solution, vortexed thoroughly, and clarified by centrifugation at 8000–10,000 rpm for 10 min at 4 °C. The supernatant was filtered through a 0.22 µm syringe filter and stored at −80 °C until inoculation. The CRFK cell line was maintained in Dulbecco’s Modified Eagle Medium (DMEM) supplemented with 10% fetal bovine serum (FBS) and 1% antibiotic-antimycotic solution at 37 °C in a humidified incubator with 5% CO_2_. Confluent monolayers grown in tissue culture flasks were washed with sterile PBS and inoculated with 0.5–1.0 mL of processed sample filtrate after removing growth medium. Following adsorption for 1 h at 37 °C with intermittent rocking, maintenance medium containing 2% FBS was added and the cultures were incubated under standard conditions. The inoculated monolayers were observed daily for CPE, characterized by cell rounding, shrinkage, increased refractility, aggregation, and detachment from the surface, typically evident within 48–72 hpi. Samples showing no CPE were subjected to three to four blind passages before being considered negative for virus isolation.

### 2.7. Sequence and Phylogenetic Analysis

The positive PCR amplified products were purified using Qiagen PCR gel purification kit (Hilden, Germany) as per the manufacturer’s instructions and sequencing was performed at M/s. Eurofins Genomics India Pvt Ltd., Bengaluru, India. The nucleotide sequence data were subjected to BLAST analysis (version 2.16.0) (www.ncbi.nlm.nih.gov), assembled and analyzed using SeqMan and MegAlign programs of Lasergene package (version 7.1.0; DNAStar Inc., Madison, WI, USA).

Nucleotide sequence alignment was performed by the ClustalW method with the MegAlign^TM^ program Version 19.0 (DNAStar Inc., Madison, Wisconsin, USA), and the predicted amino acid sequences were analyzed by the Protean^TM^ program of Lasergene version 19.0 (DNAStar Inc., Madison, WI, USA).

Phylogenetic analysis of FPV isolates of this study, Indian, vaccine, and other country FPV strains were performed based on the VP2 gene region using the maximum likelihood method with 1000 bootstrap replications in the MEGA software version 12.

### 2.8. B-Cell Epitope Prediction

The FPV field strains from this present study were compared with vaccine and other country strains to assess variation in predicted B-cell epitopes. B cell epitope prediction was performed using the Immune Epitope Database and Analysis Resource (IEDB) online platform.

### 2.9. Selection Pressure Analysis

Selection pressure acting on the VP2 gene was assessed by estimating the ratio of nonsynonymous (dN) to synonymous (dS) substitution rates (ω = dN/dS). Analyses were performed using the Fixed Effects Likelihood (FEL) method implemented in the Datamonkey 2.0 web server (http://www.datamonkey.org accessed on 12 March 2026). Sites were classified as under purifying selection (ω < 1) or diversifying selection (ω > 1) based on statistical significance (*p* < 0.05).

### 2.10. Pairwise Genetic Distance Calculation

Pairwise genetic distances between sequences were calculated using the Jukes-Cantor (JC) model in MEGA12 to quantify nucleotide divergence among field isolates, and between field isolates and the vaccine reference strain. Distances were reported as percentages of base substitutions per site.

### 2.11. Structural Analysis of TfR1-VP2 Interactions

To investigate the molecular basis of receptor recognition, structural analyses were performed using the TfR1 protein structures for feline and canine hosts. Three-dimensional structures of feline and canine TfR1 were retrieved from the AlphaFold Protein Structure Database (AlphaFold DB) as PDB files [22]. Structural alignment and comparative analysis were conducted to identify species-specific residues in the apical domain that may influence VP2 binding affinity and host range determination. Three-dimensional structures of VP2 from FPV and CPV were retrieved from the RCSB Protein Data Bank (PDB) website.

### 2.12. Molecular Docking

Molecular docking was performed using BIOVIA ZDOCK to predict the binding conformations and interaction interfaces between VP2 and TfR1. Docking poses were analyzed to identify key residues involved in receptor binding. The top-ranked pose by ZDOCK score was selected, and its ZRANK score was calculated to estimate binding stability. Comparative analysis between feline and canine complexes was performed to assess differences in binding affinity and interaction patterns.

### 2.13. Statistical Analysis

Descriptive statistics were used to summarize FPV PCR positivity, clinical signs, specimen types, and epidemiological variables. Associations between sex, age group, breed group, vaccination status, and season with FPV PCR positivity were evaluated using multivariable binary logistic regression. Adjusted odds ratios (aORs) with 95% confidence intervals (CIs) were calculated, and *p* < 0.05 was considered statistically significant. Model convergence, coefficient stability, and multicollinearity were assessed before interpretation. Statistical analyses were performed using R software version 4.4.3 (Accessed on 21 April 2026).

## 3. Results

### 3.1. Clinical Cases

A total of 180 domestic cats presenting with clinical signs suggestive of feline panleukopenia were included in the study.

### 3.2. Epidemiological Pattern

#### 3.2.1. Prevalence of Feline Panleukopenia Virus

**Figure 1:**
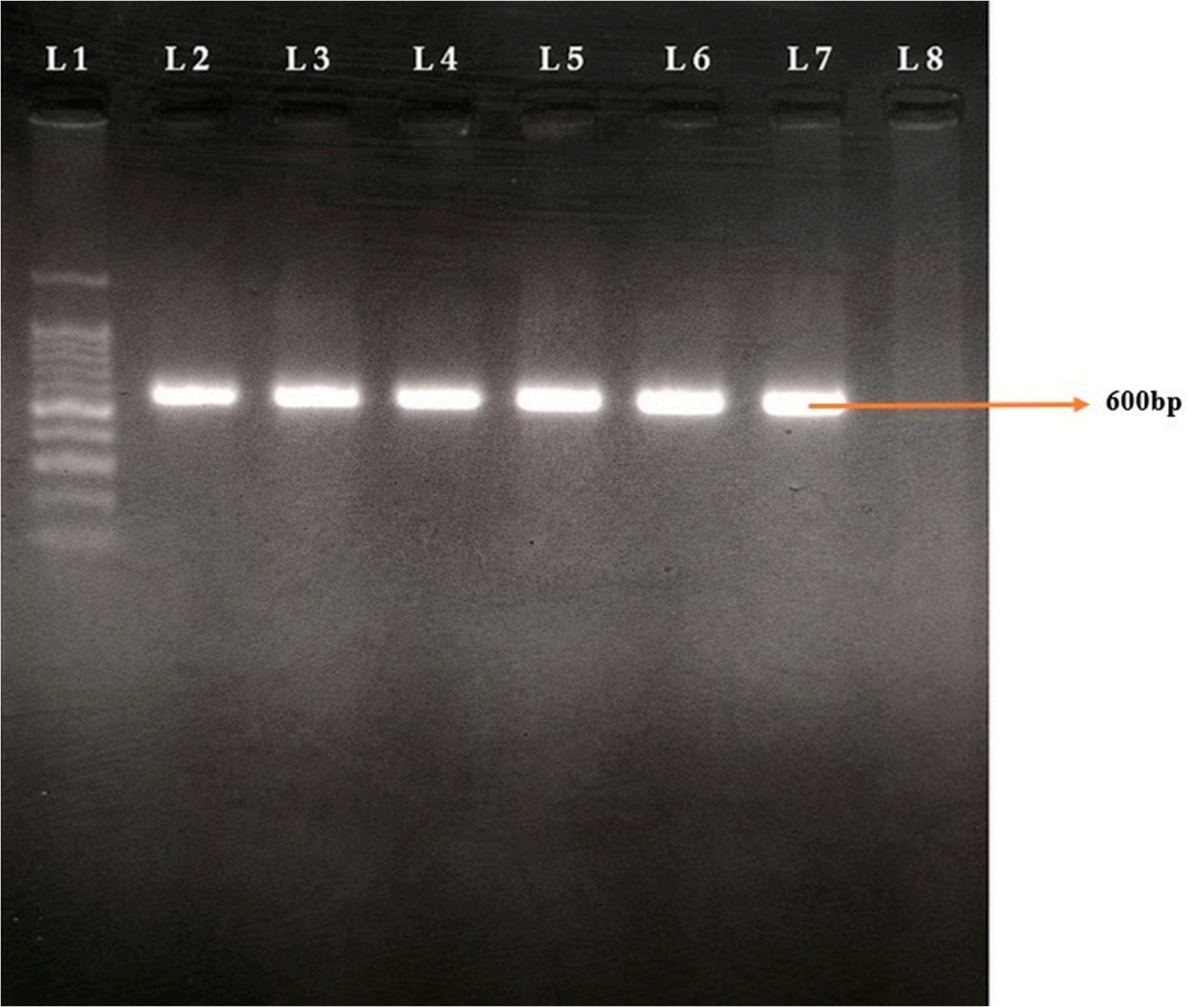
PCR amplification of feline panleukopenia virus. Lane 1: Marker (100 bp), Lanes 2–6: suspected FPV samples, Lane 7: positive control, and Lane 8: negative control.

The prevalence of feline panleukopenia virus infection was 46.11% (83/180) among clinically suspected cats during the one-year study period, based on PCR amplification of the VP2 capsid protein (Figure 1). Among the 83 positive samples, mixed infection were detected in 3.6% (03/83 cats) of cases involving FHV and FPV, and in 1.2% (01/83) of cases involving FCoV and FPV. During the study period, the case fatality rate among FPV-positive cats was 7.23% (06/83). Further, among the six deceased cats, the possibility of the mixed infection was 16.66% (01/06) for feline panleukopenia, feline herpesvirus, feline leukemia virus, and feline calicivirus infections, as confirmed by PCR.

#### 3.2.2. Clinical Specimen Collection and Animal-Level PCR Positivity

Among cats from which each specimen type was collected, animal-level PCR positivity was 46.8% (81/173) for fecal swabs, 45.5% (5/11) for oral swabs, 31.6% (12/38) for conjunctival swabs, 33.3% (2/6) for blood, 50.0% (1/2) for ascitic fluid, and 100% (1/1) for nasal swabs. These proportions represent the overall PCR status of the animals and not the PCR positivity of the individual specimen types, therefore, no diagnostic performance comparison was made.

#### 3.2.3. Clinical Presentation of FPV-Positive Cats

**Figure 2:**
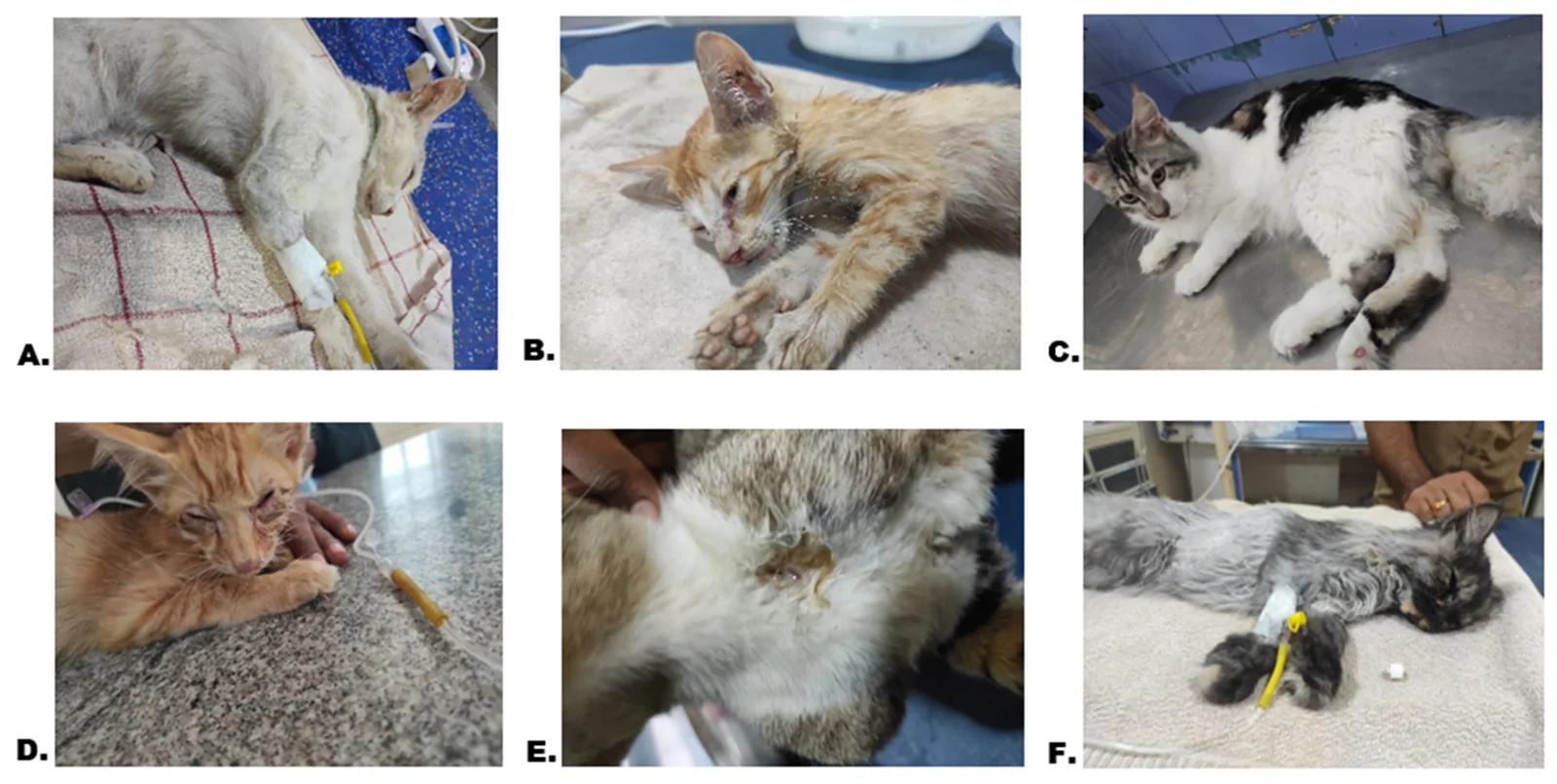
Clinical signs observed in the feline panleukopenia suspected cats. (**A**–**C**) Severe dehydration. (**D**) Diarrohea. (**E**) Anorexia. (**F**) Leukopenia.

Among the 83 FPV PCR-positive cats, diarrhoea, anorexia, and dehydration were each recorded in 73 cats (87.95%), while vomiting was observed in 72 cats (86.75%). The distribution of the recorded clinical signs is shown in Figure 2. Clinical signs were summarized descriptively and were not considered epidemiological risk factors.

#### 3.2.4. Epidemiological Factors Associated with FPV PCR Positivity

**Figure 3:**
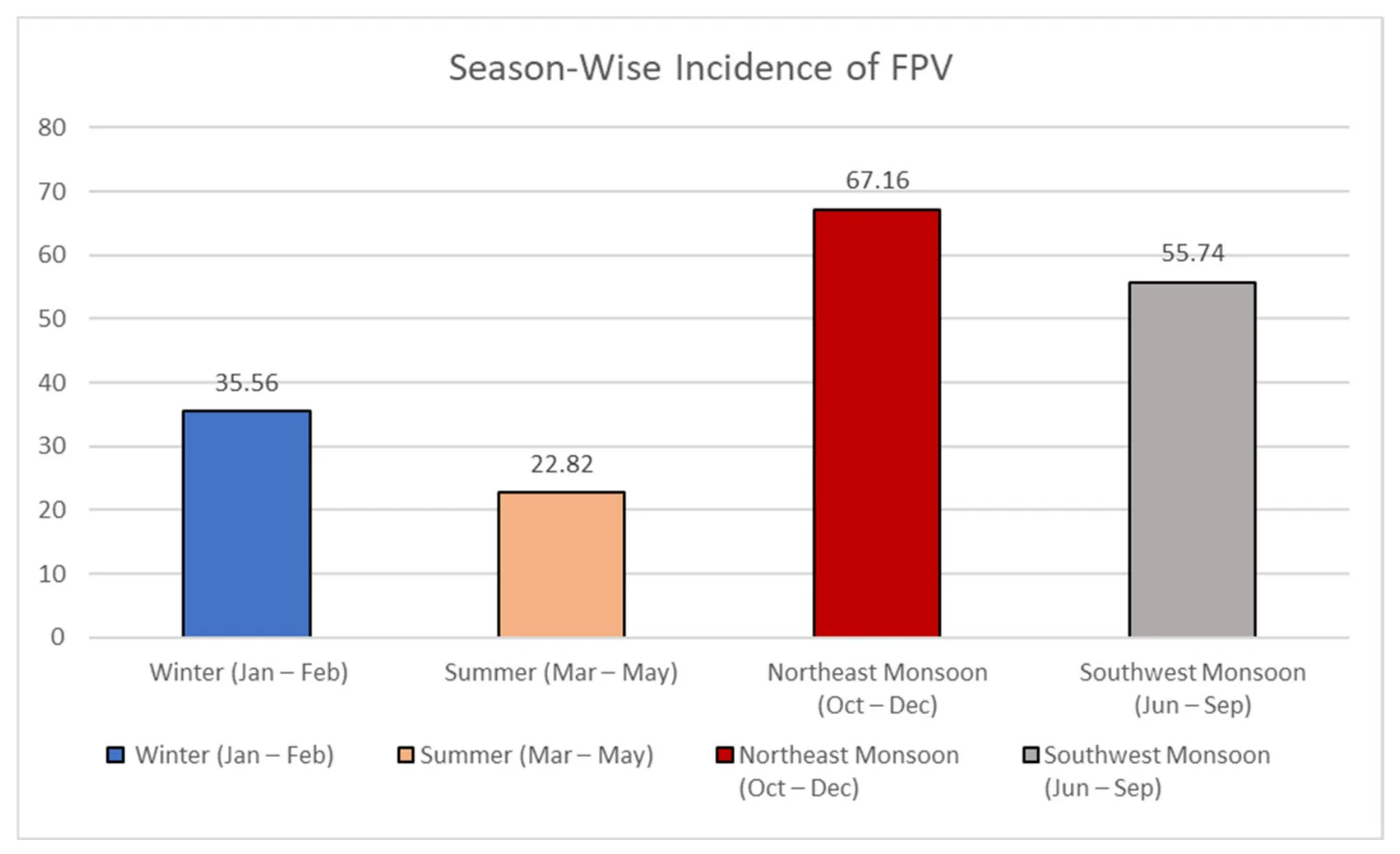
Season-wise occurrence of FPV.

**Figure 4:**
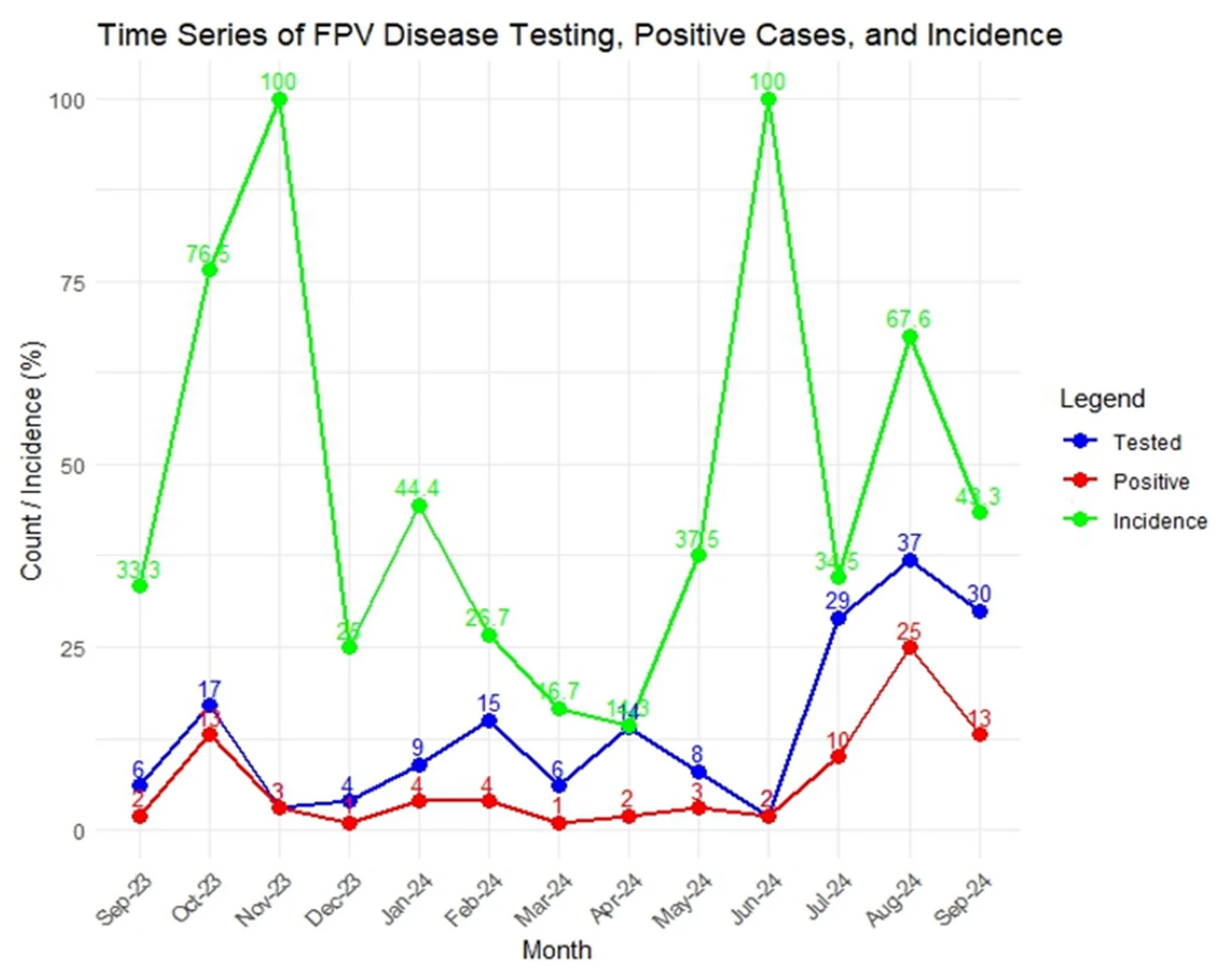
Month-wise occurrence of FPV.

The associations of sex, age group, breed, vaccination status, and season with FPV PCR positivity were evaluated using multivariable logistic regression (Table 1). Female cats had significantly higher odds of PCR positivity than male cats (adjusted odds ratio [aOR]: 2.65; 95% confidence interval [CI]: 1.35–5.21; *p* = 0.0047). Vaccinated cats had significantly lower odds of PCR positivity than unvaccinated cats (aOR: 0.41; 95% CI: 0.17–0.97; *p* = 0.0429). Compared with summer, the odds of FPV PCR positivity were significantly higher during the southwest monsoon (aOR: 3.59; 95% CI: 1.26–10.20; *p* = 0.0165) and northeast monsoon (aOR: 17.11; 95% CI: 3.86–75.80; *p* < 0.001). No significant association was observed for winter (*p* = 0.6127). Age group and breed were not significantly associated with FPV PCR positivity. The descriptive seasonal and month-wise distributions of FPV PCR positivity are presented in Figure 3 and Figure 4, respectively.

### 3.3. Gross Pathological Lesions

**Figure 5:**
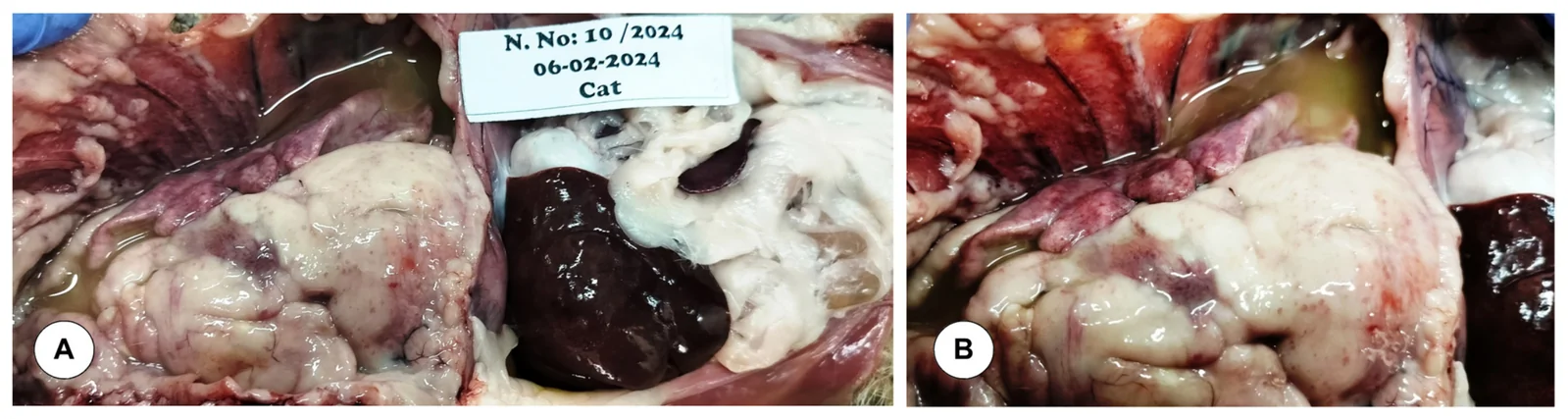
Gross pathological lesions in FPV-infected cats. (**A**) Thoracic cavity contained fibrinous exudate, pale and congested lungs with petechial hemorrhages. Abdominal cavity contained markedly enlarged, congested and hemorrhagic liver with atrophy of spleen. (**B**) Higher magnification of (**A**).

Gross pathological lesions observed in cats infected with FPV were severe dehydration with sunken eye balls, emaciation, pale anemic conjunctival and oral mucous membranes, and ocular and nasal discharges. Small intestine especially jejunum and ileum were diffusely distended with gas, catarrhal, fibrinous, and hemorrhagic contents. Intestinal mucosa was congested, edematous, and thickened with necrotic debris. Petechial and ecchymotic hemorrhages were observed on intestinal serosa and mucosa. Mesenteric lymph nodes were enlarged, congested and edematous, and in some cases marked atrophy. Lymphoid organs like thymus, spleen, and peripheral lymph nodes (prescapular, mandibular, inguinal, and popliteal) showed atrophy. Bone marrow was pale and depleted. Lungs showed congestion and edema with hydothorax and ascites in some cases. Markedly enlarged, congested and hemorrhagic liver (Figure 5A,B).

### 3.4. Virus Isolation in CRFK Cell Line

**Figure 6:**
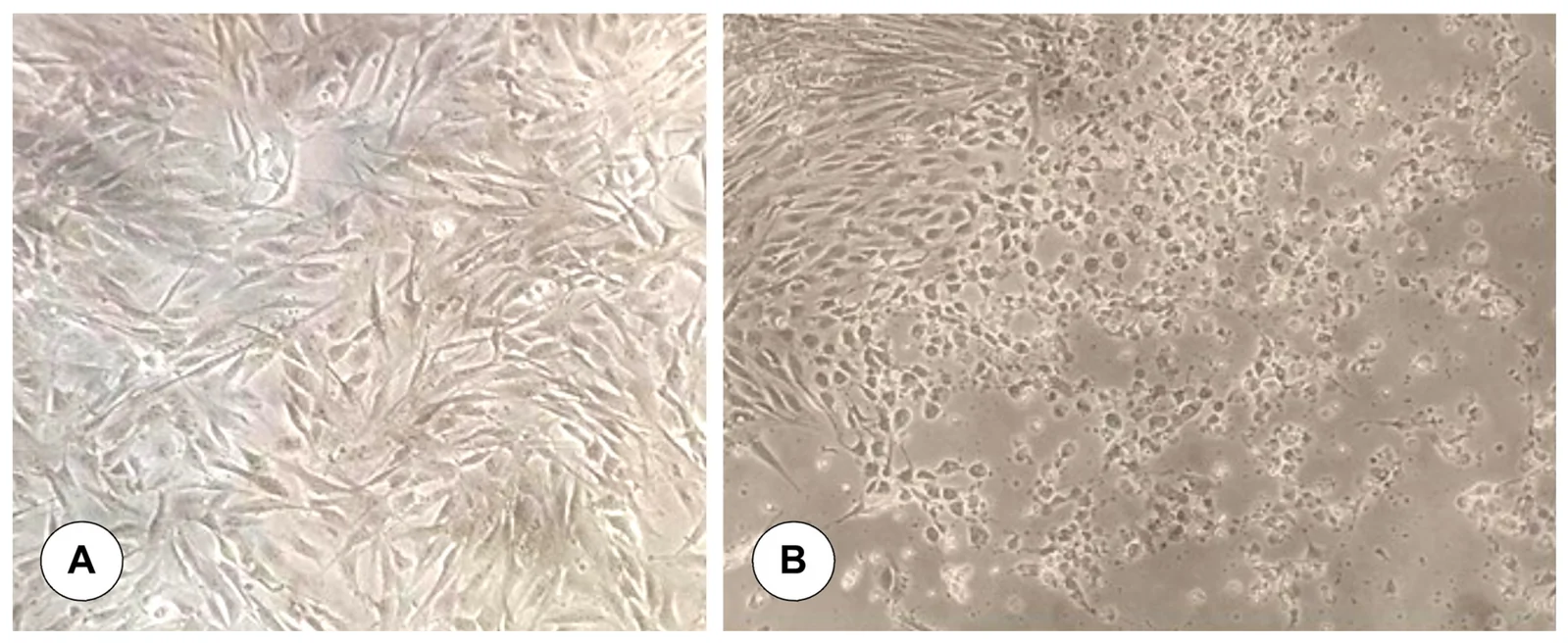
Feline panleukopenia virus-induced cytopathic effects in CRFK cell line. (**A**) Uninfected cells showed adherent compact spindle- to fusiform-shaped morphology. (**B**) FPV-infected cells showed cell rounding, detachment, cytoplasmic vacuolation, granularity, and syncytium formation.

Ten FPV-positive field samples were subjected to virus isolation in the CRFK cell line. Following inoculation, infected monolayers began to exhibit characteristic cytopathic effects (CPEs) within 48–72 hpi, initially characterized by focal areas of cell rounding and increased refractility. As infection progressed, extensive cell shrinkage, aggregation, and detachment from the culture surface were observed, leading to partial or complete destruction of the monolayer upon subsequent passages (Figure 6B). The intensity and onset of CPE became more pronounced after the second- and third-blind passages, indicating successful adaptation of the virus to in vitro conditions. Of the 10 samples subjected to virus isolation, four isolates were successfully recovered and propagated in CRFK cells. Uninfected control CRFK cells remained healthy and confluent without morphological alterations (Figure 6A). The consistent development of characteristic parvoviral CPEs in the recovered isolates confirmed successful replication of FPV in the CRFK cell line.

### 3.5. Molecular Characterization of Feline Panleukopenia Strains

Among 83 positive samples, 13 FPV-positive samples and one Feligen^®^ CRP/L vaccine (Virbac Animal Health India Pvt Ltd., Mumbai, Maharashtra, India) were selected based on different breeds, age, severity of infections, vaccination status, and sampling location for sequencing by targeting VP2 capsid gene region from 2822 to 3500 bp, and submitted to GenBank with the accession numbers: PP783785 to PP783791 and PQ495725 to PQ495730 for FPV samples and PQ495731 for vaccine. BLAST analysis revealed that all the VP2 genome sequences of field FPV strains shared 99.07–99.53% nucleotide identity with FPV strains of different countries. The results were shown in Table 2.

**Figure 7:**
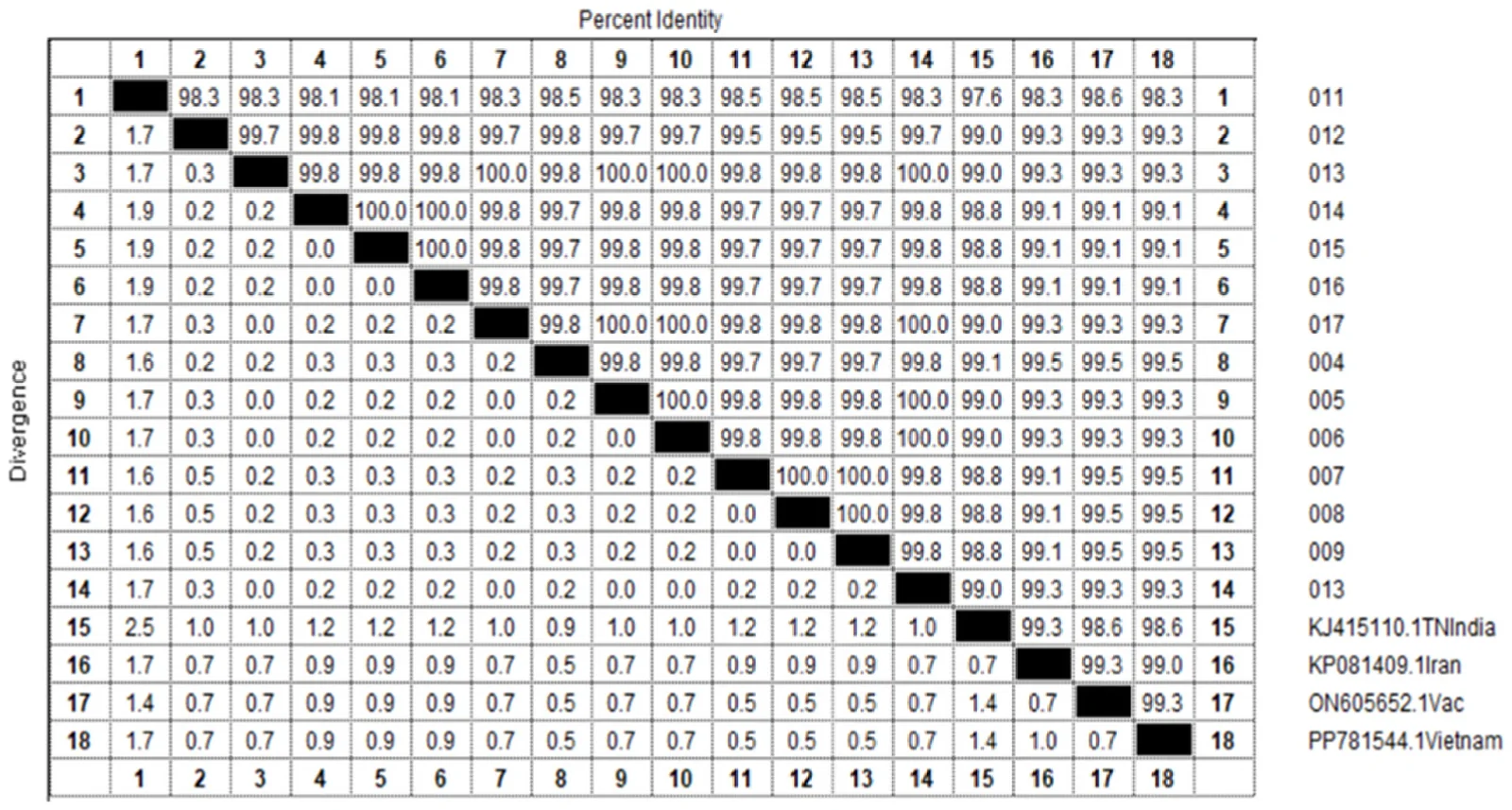
Homology analysis of FPV field, vaccine, and other country strains.

The VP2 genome sequence of field FPV strains shared 98.1–98.5% nucleotide identity with vaccine strains and divergence was 1.6–1.9%. The field FPV strains showed the percentage identity ranged from 99.5 to 100%, and the divergence was 0.5%. The percentage of identity between the FPV strains sequenced from this study and the sequences retrieved from India (KJ415110), other country vaccines (ON605652), and FPV field strains (Iran and Vietnam) was found to be between 98.8 and 100%, and divergence was 1.2% (Figure 7).

#### 3.5.1. Nucleotide Variations in Feline Panleukopenia Virus

**Figure 8:**
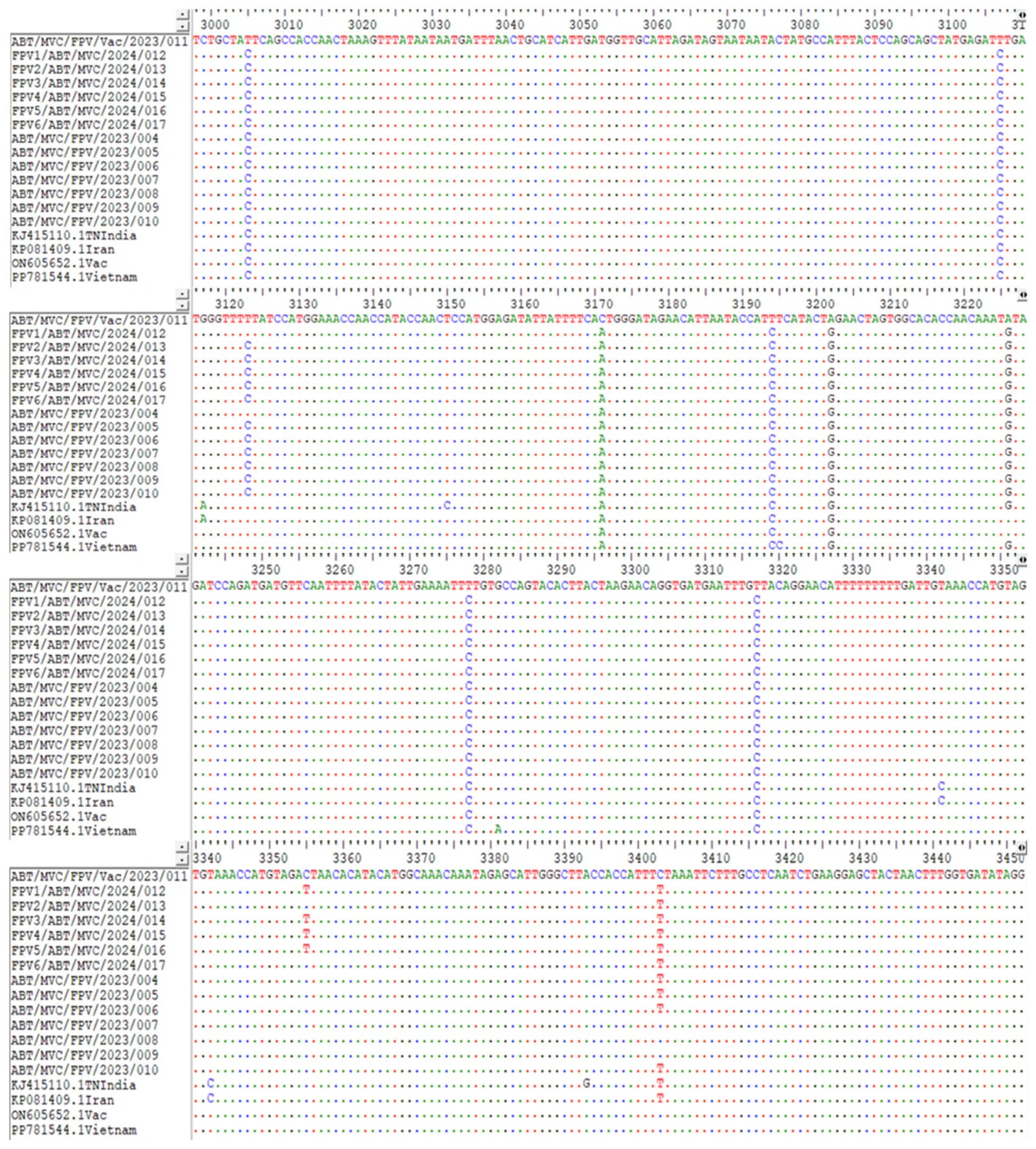
Comparison of nucleotide variations in the FPV-positive field samples with Indian FPV and other country strains.

The nucleotide variations among 13 FPV field strains, Indian (KJ415110), other country vaccines (ON605652), and FPV field strains (Iran and Vietnam) were compared at VP2 target region and depicted in Table 3 and Figure 8.

The FPV VP2 capsid protein gene revealed seven unique nucleotide variations at positions 3005 (T-C), 3107 (T-C), 3278 (T-C), 3317 (T-C), 3342 (T-C), 3355 (C-T), and 3403 (C-T) comparing with vaccine strains. While comparing with other Indian strains, among seven nucleotide variations, six were the same as mentioned above except at position 3355 (C-G). Interestingly, most of the nucleotide changes identified were T-C substitution.

#### 3.5.2. Amino Acid Variations in Feline Panleukopenia Virus

**Figure 9:**
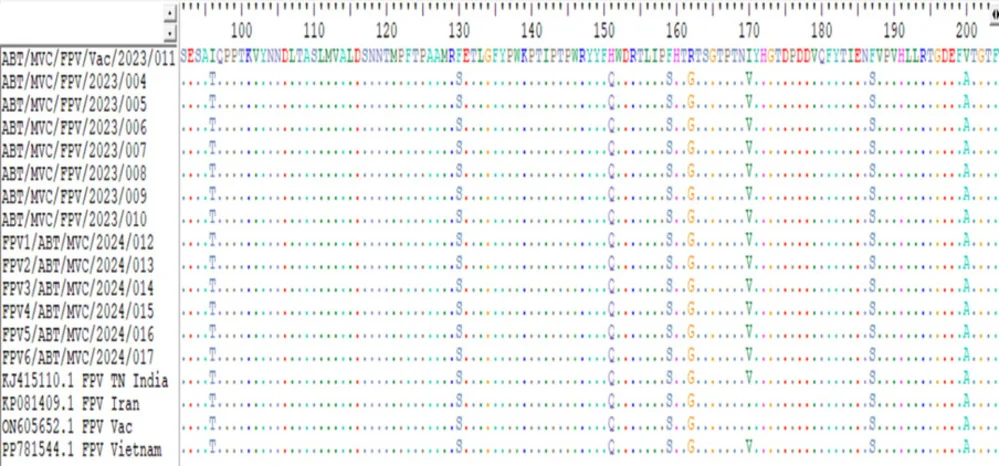
Comparison of amino acid variations in the FPV samples with Indian FPV and other country strains.

The deduced amino acid sequences of the FPV strains identified in the present study were compared with Indian FPV strain (KJ415110), other country vaccine strain (ON605652), and FPV field strains reported from other countries such as Iran and Vietnam. The amino acid variations identified within the VP2 region are presented in Table 4 and Figure 9.

Eight unique amino acid variations were observed between the FPV field strains, vaccine strain and FPV strains of Indian and International FPV strains at positions Ile96Thr, Phe130Ser, His151Cys, Phe159Ser, Arg162Gly, Ile170Val, Phe187Ser, and Val200Ala. The amino acid sequences were most likely similar between the present study FPV strains and the vaccine strain currently used in India.

#### 3.5.3. Phylogenetic Analysis of FPV Strains

**Figure 10:**
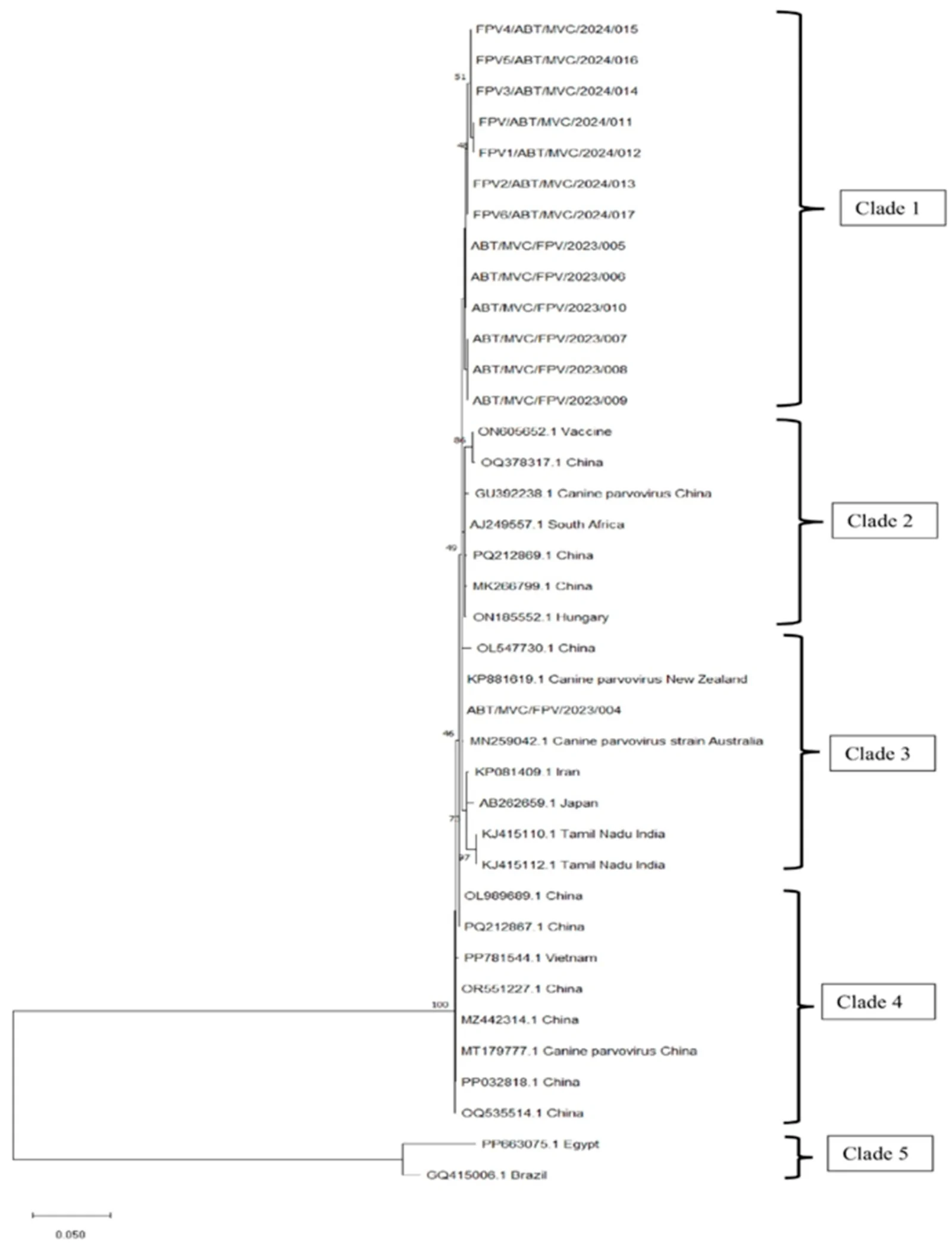
Phylogenetic analysis of partial VP2 gene sequencing of FPV-positive stains with Indian and other country FPV strains using maximum likelihood method with 1000 bootstrap replicates.

The phylogenetic analysis revealed five distinct clades among the FPV strains analysed (Figure 10). Clade 1 consisted of 12 FPV strains identified in the present study along with the vaccine strain currently used in India. Clade 2 comprised of FPV vaccine strains (ON605652), FPV strains from China, South Africa, and Hungary along with canine parvovirus strain from China. Clade 3 included FPV strains from other countries (China, Iran and Japan), Indian FPV strains (Tamil Nadu strains), and canine parvovirus strains from Australia and New Zealand. One FPV stain from the present study forms a cluster with FPV and CPV strains in clade 3, which denoted the close relatedness of FPV and CPV strains in India. Clade 4 consists of FPV strains majorly from China and one CPV strain and FPV strain from Vietnam. Clade 5 consisted of FPV strains from Egypt and Brazil, which differed from all other FPV strains reported earlier from throughout the world.

#### 3.5.4. B-Cell Epitope Prediction of FPV Strains

**Figure 11:**
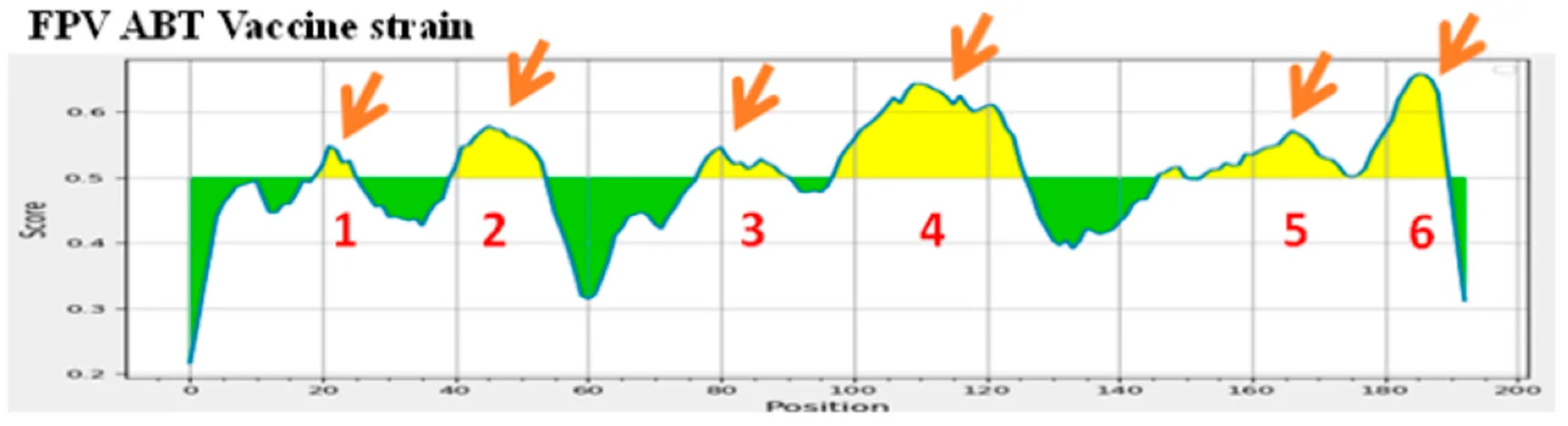

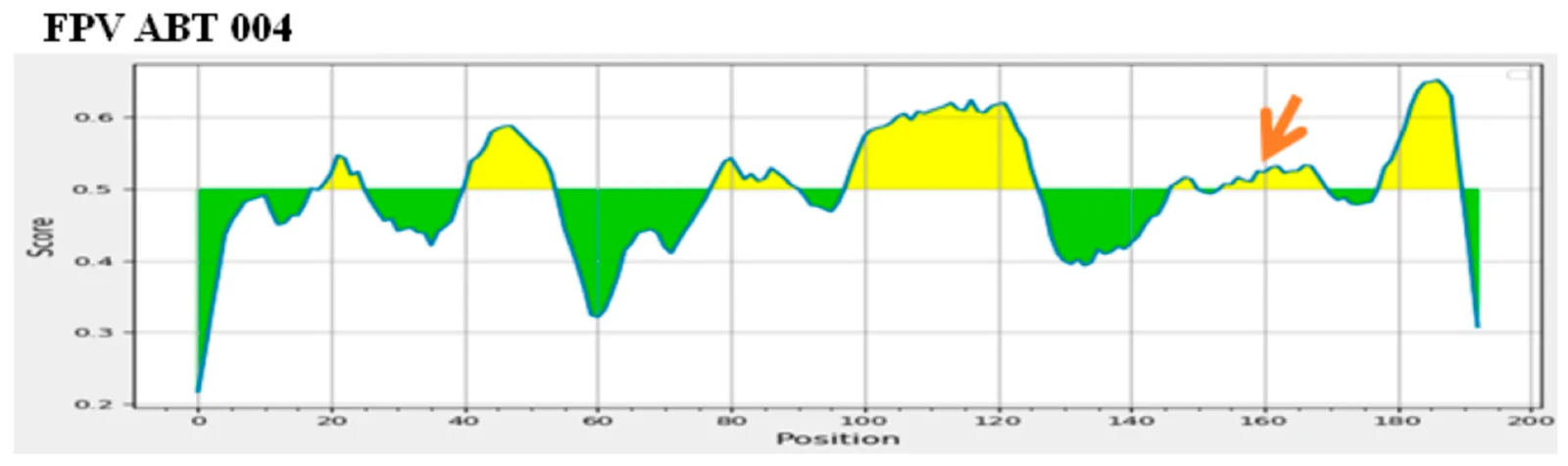

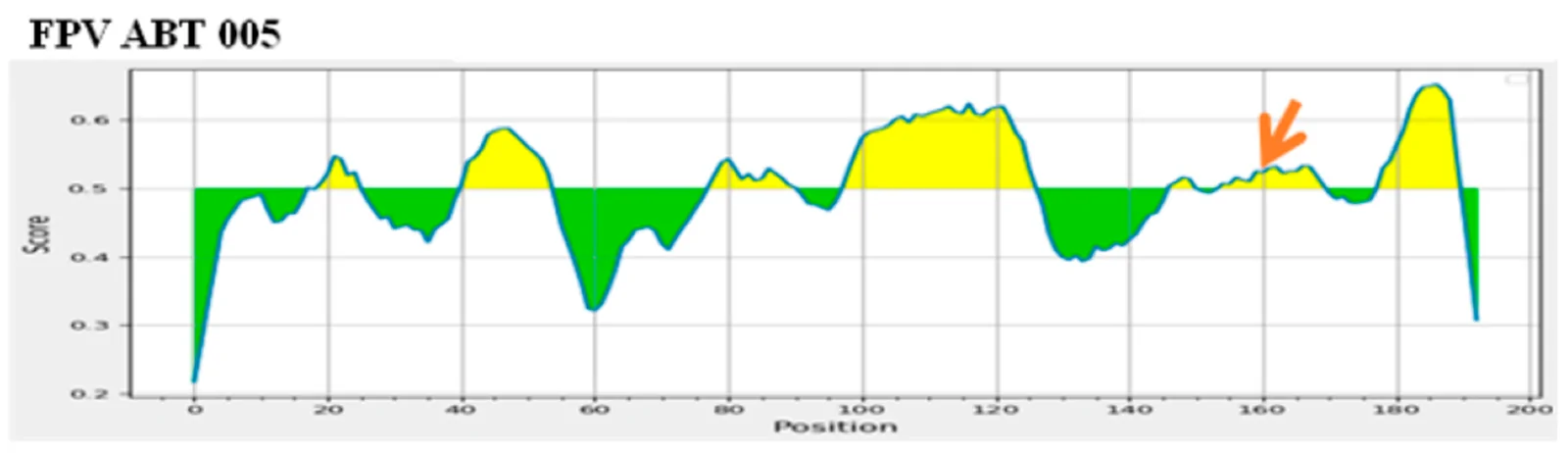

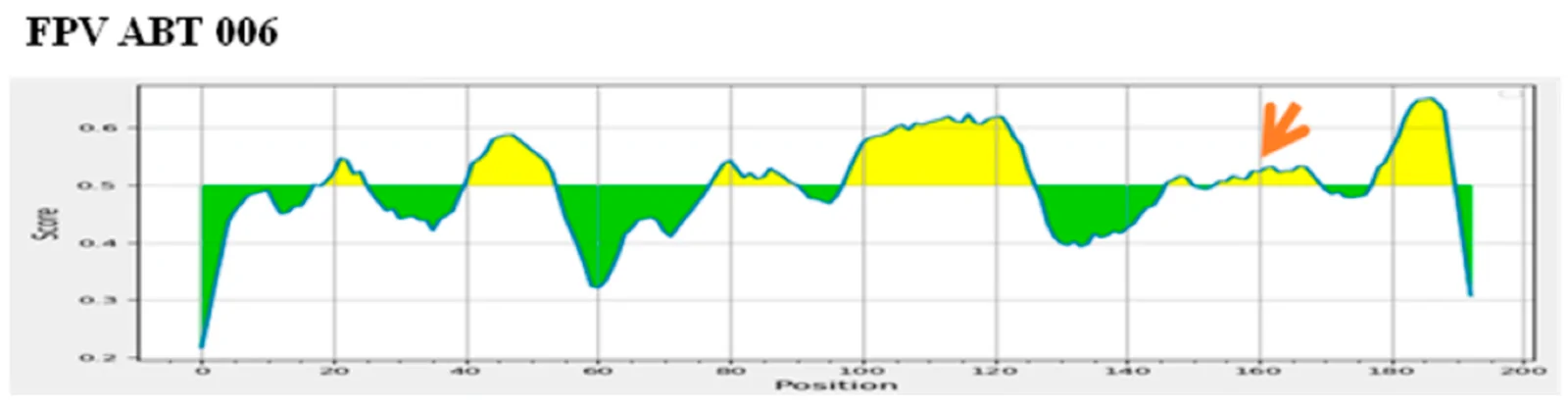

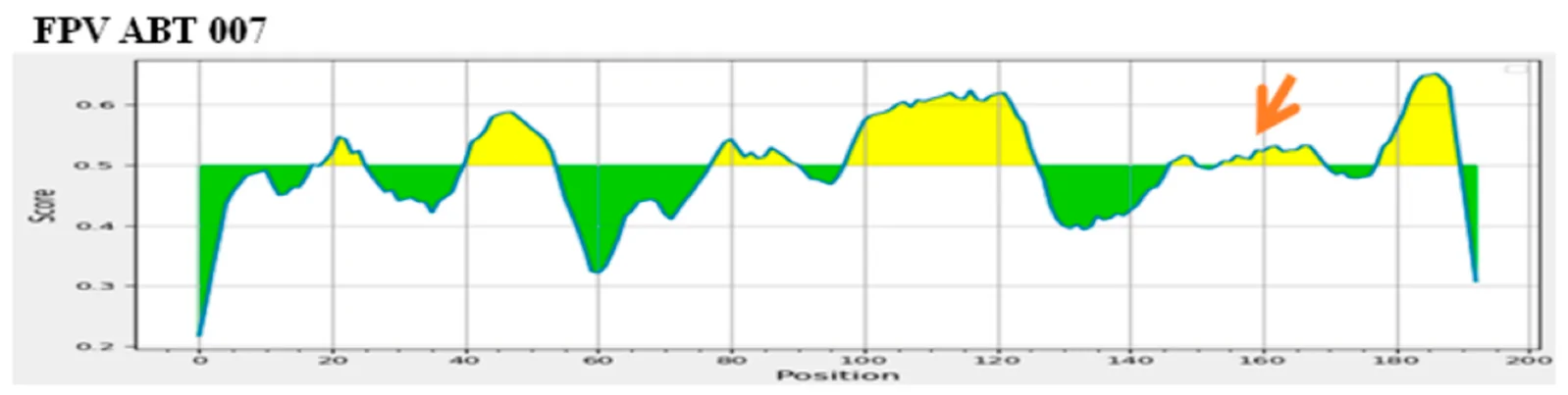

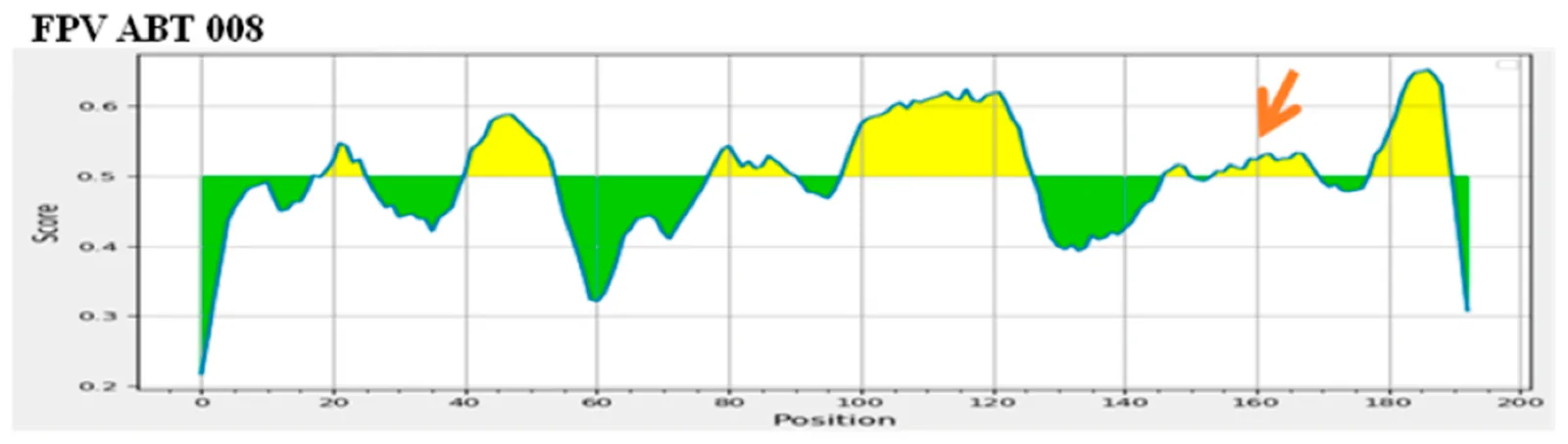

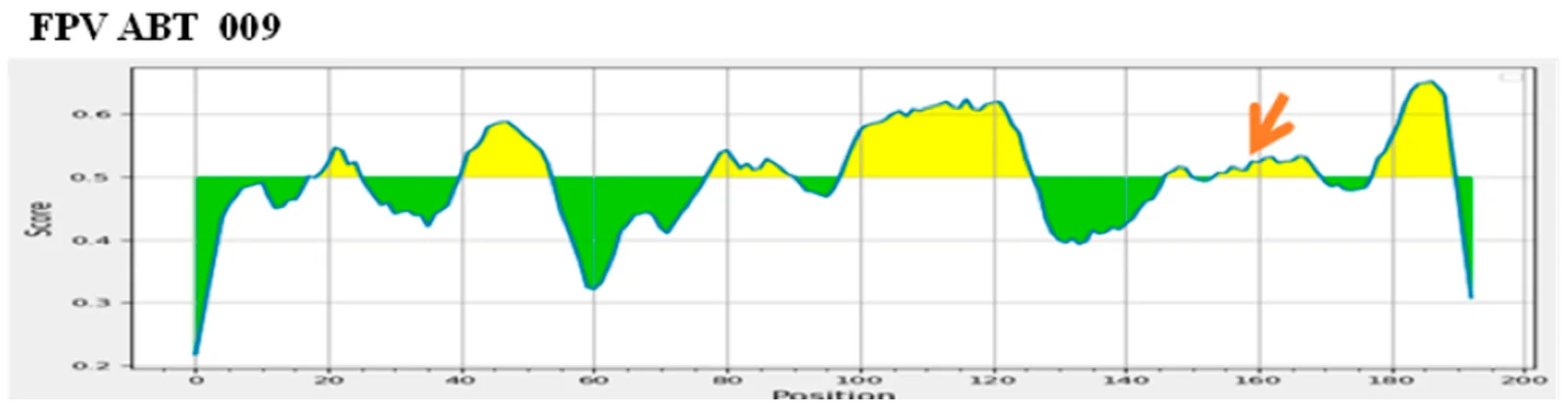

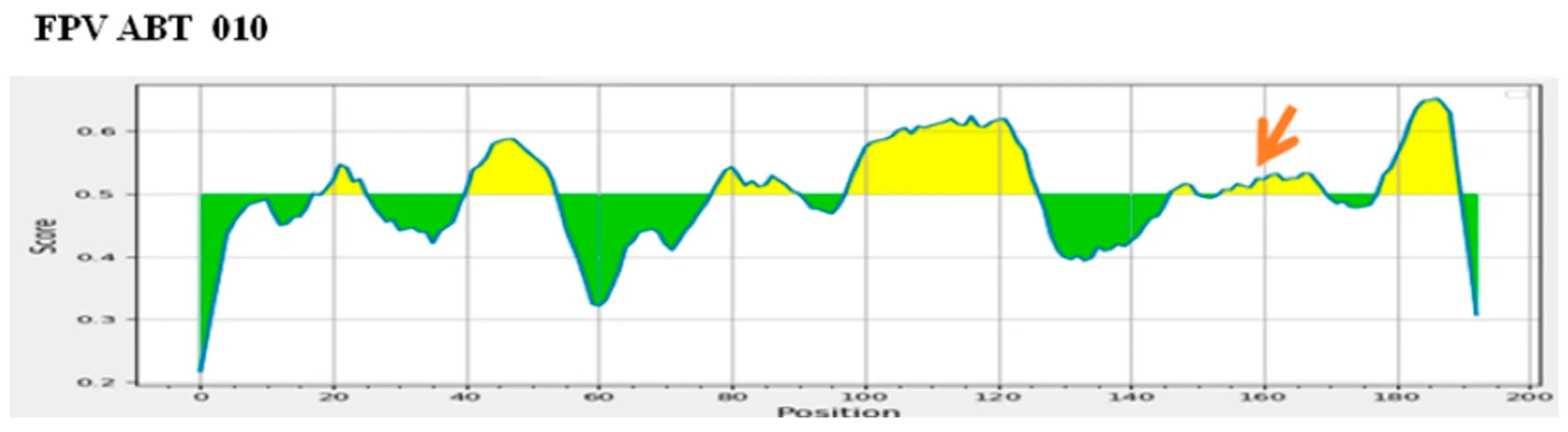

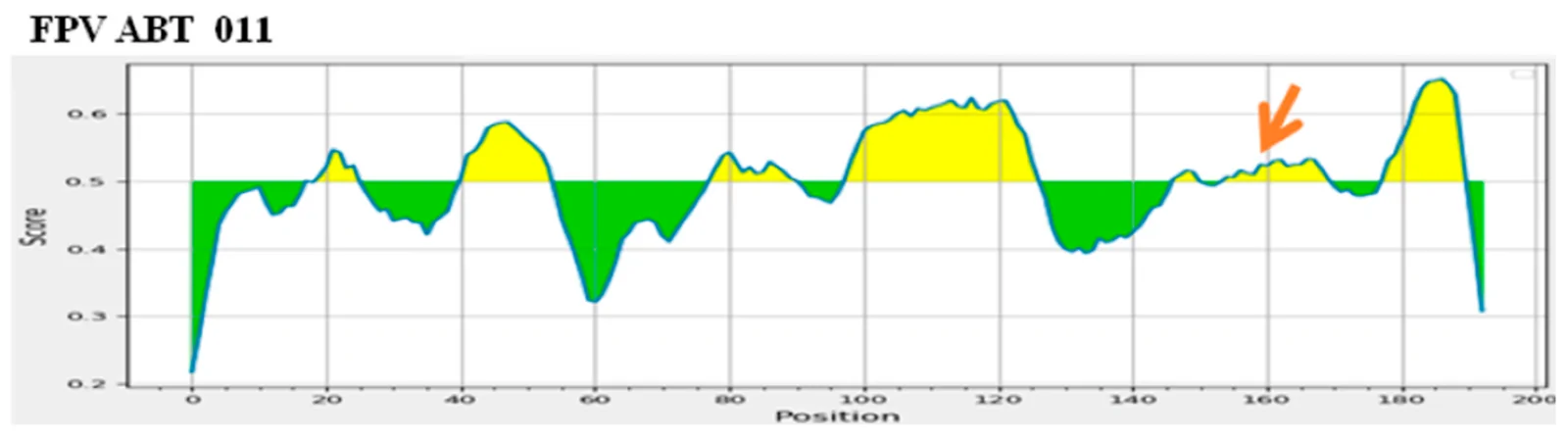

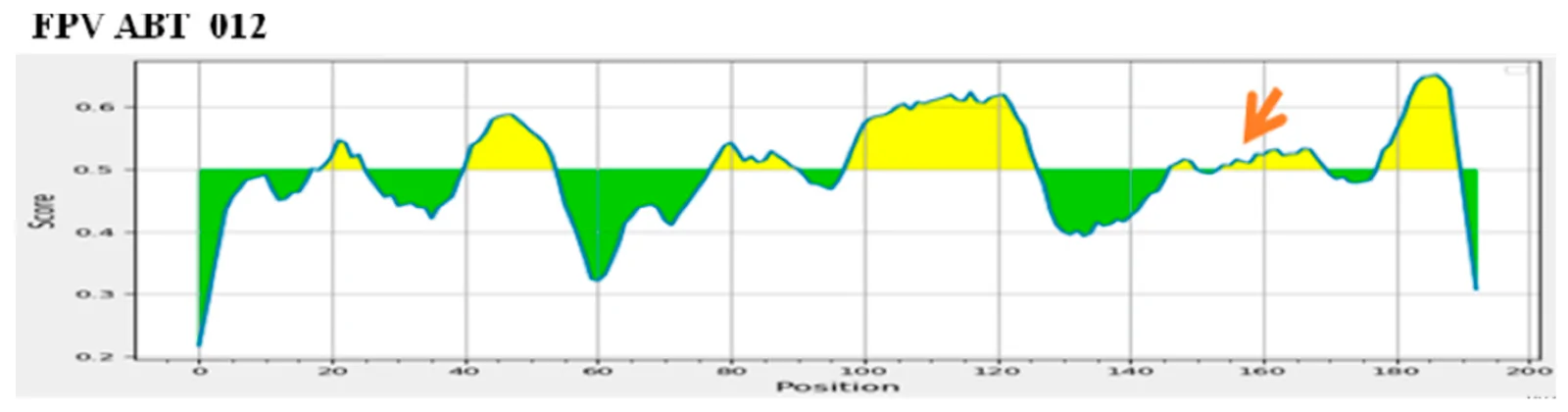

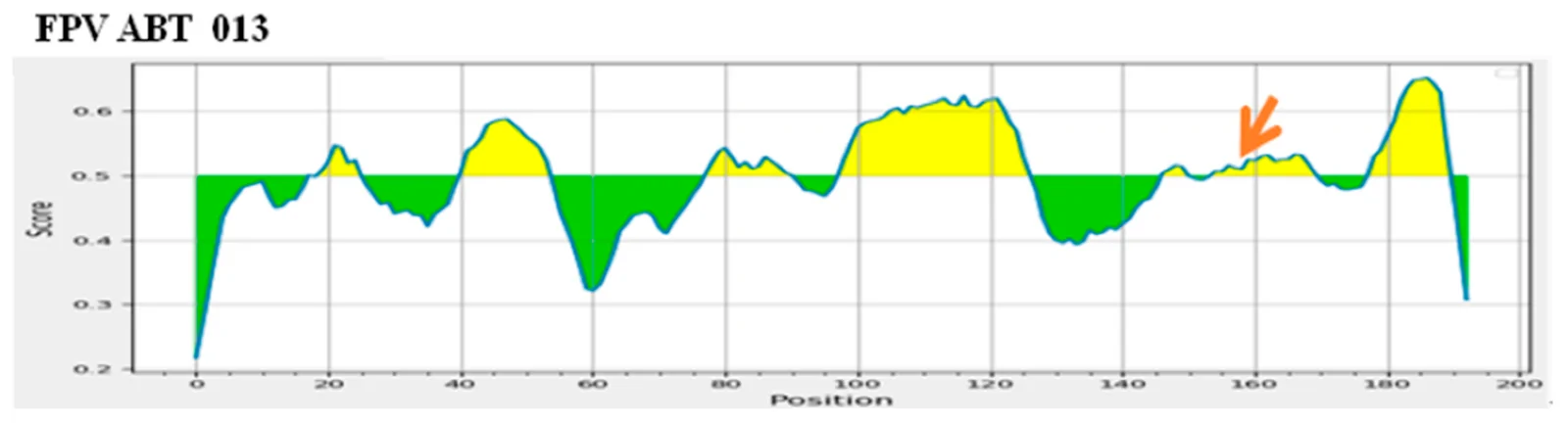

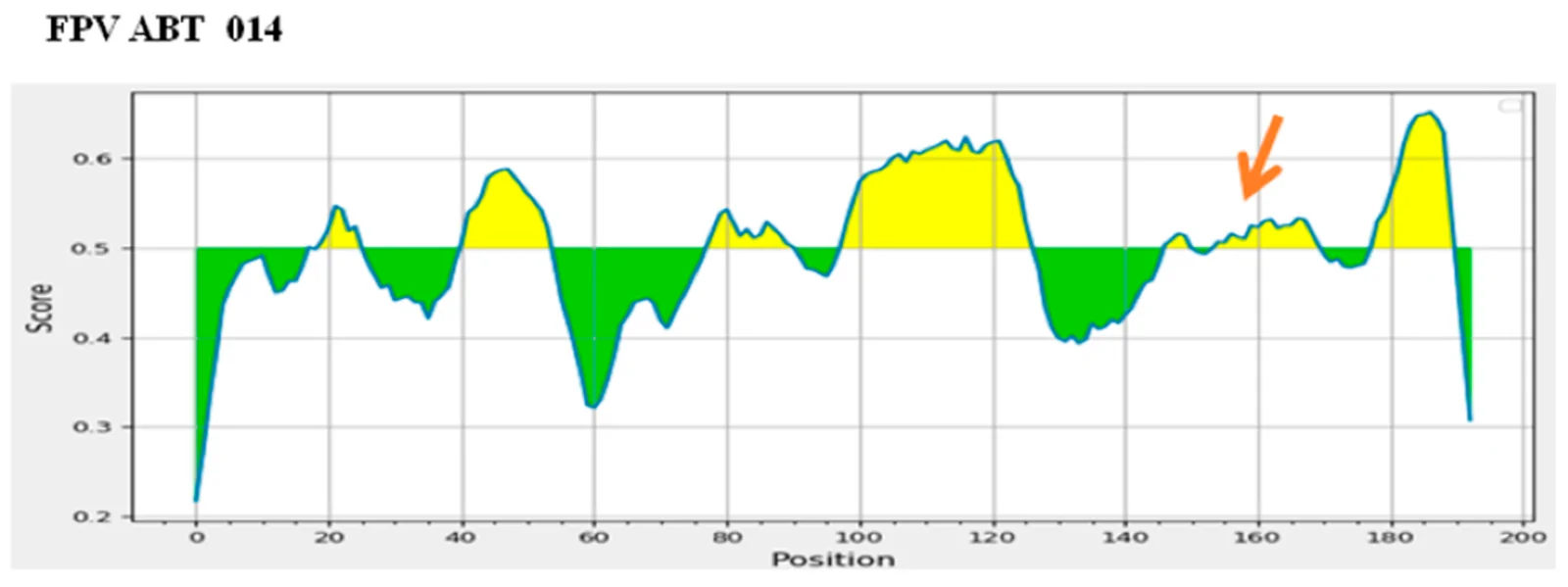

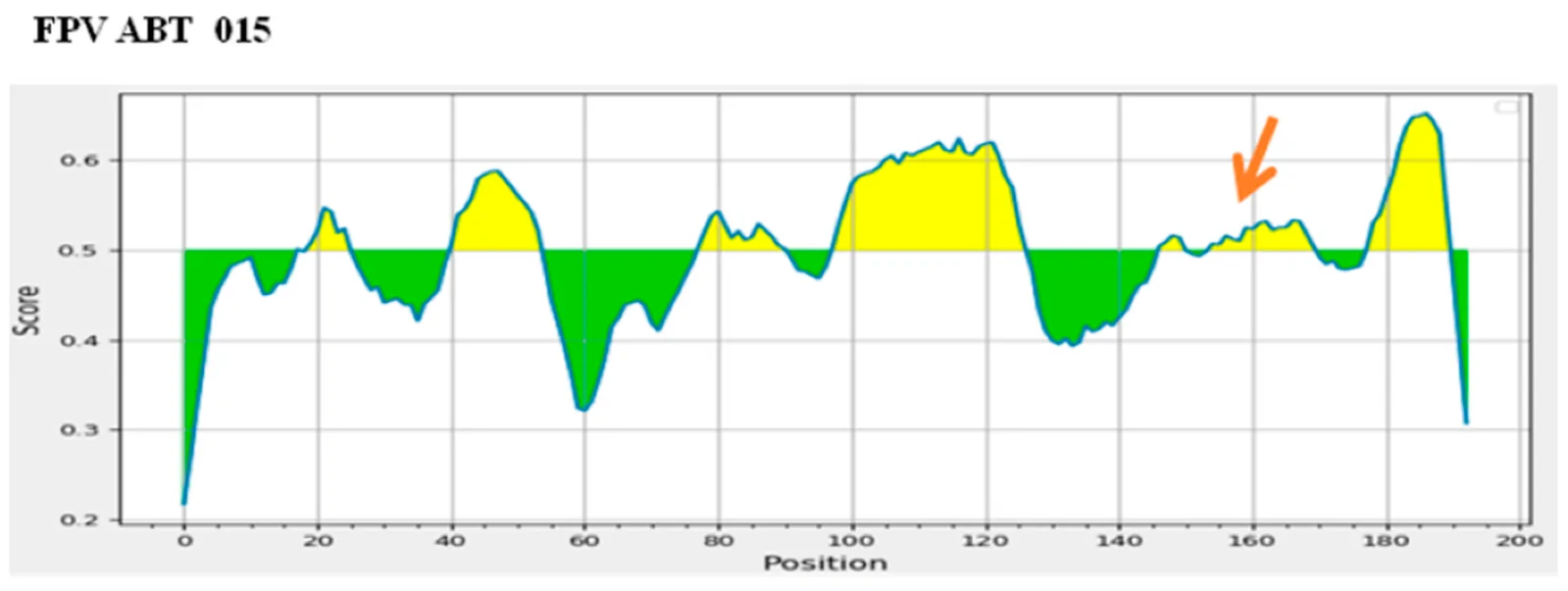

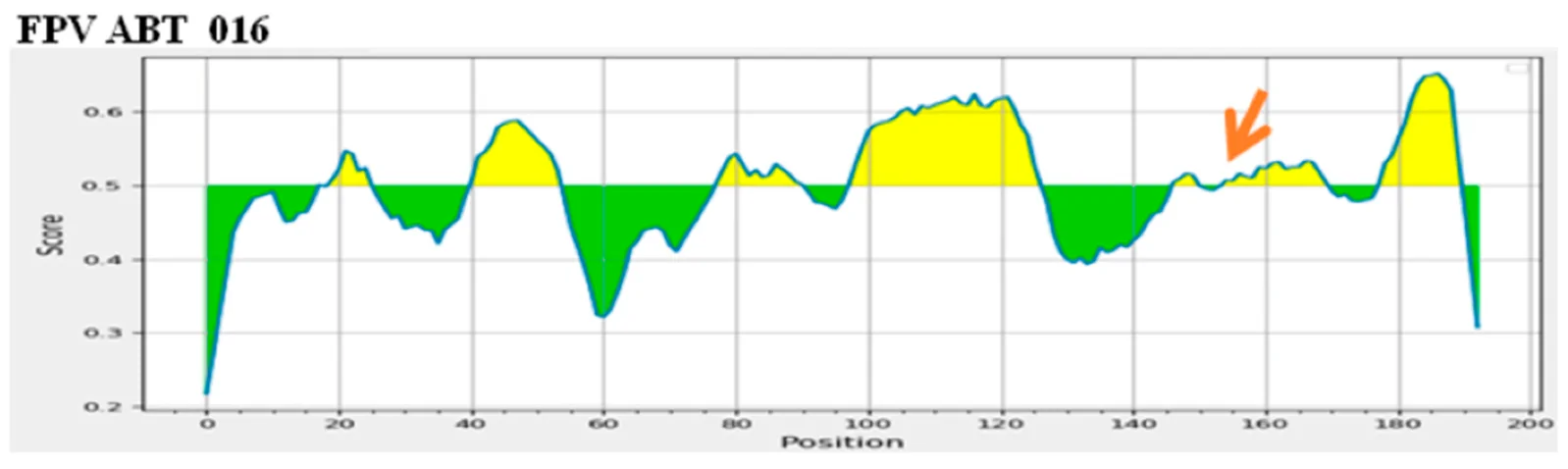

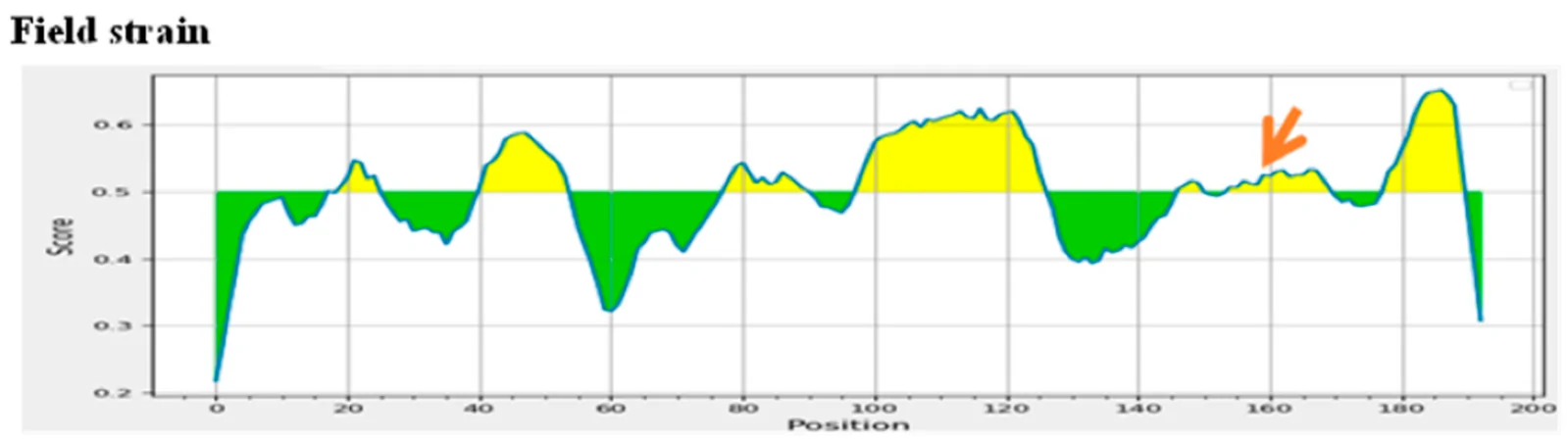
B-cell epitope prediction for FPV field, vaccine, and other country strains.

**Figure 12:**
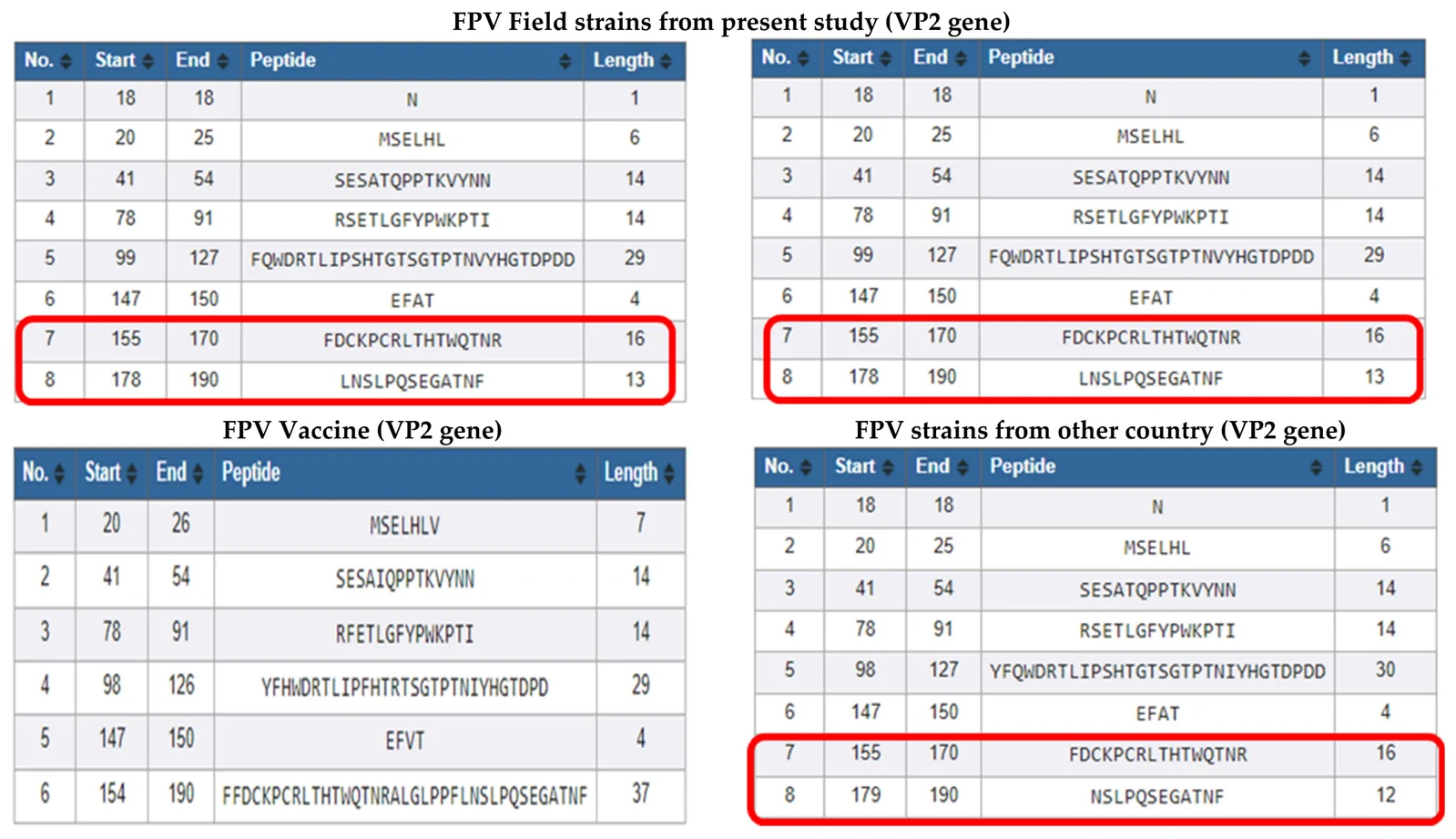
Amino acids sequences predicted by B-cell epitope prediction software in the field, vaccine, and other country FPV strains in the antigenic determinant regions of VP2.

Predicted B-cell epitopes within the antigenic determinant region of the VP2 protein differed between the field and vaccine strains. A missing peak at the fifth position was observed in the field strains compared with the vaccine strain, reflecting the genetic variations identified in the VP2 gene. The total number of predicted peptides within the antigenic determinant region was eight in the field strains and six in the vaccine strain (Figure 11 and Figure 12). These differences suggest variation in the predicted antigenic profiles of the analyzed strains, however, their biological significance remains uncertain and requires experimental validation through immunological assays.

#### 3.5.5. Selection Pressure Analysis

Selection pressure analysis revealed distinct patterns of evolutionary constraints across the VP2 gene. Five sites (175, 178, 179, 190, and 232) were identified as being under purifying selection (*p* < 0.05), with alpha values substantially greater than beta values, indicating strong functional constraints at these positions. Conversely, two sites (45 and 152) showed evidence of diversifying selection (*p* < 0.05), suggesting potential adaptive evolution at these residues. However, as the analysis was performed using only 13 VP2 sequences, these findings should be interpreted with caution and require validation using larger datasets.

Detailed site-by-site results from the Fixed Effects Likelihood (FEL) analysis are presented in Table 5.

#### 3.5.6. Pairwise Genetic Distances

Pairwise genetic distances among the field isolates ranged from 0.15 to 0.26%, indicating a high degree of sequence conservation within the circulating field population. In contrast, the genetic distance between the field isolates and the vaccine strain was substantially higher, ranging from 2.18 to 2.72%, suggesting moderate genetic differentiation.

### 3.6. Docking Analysis of TfR1-VP2 Complexes

**Figure 13:**
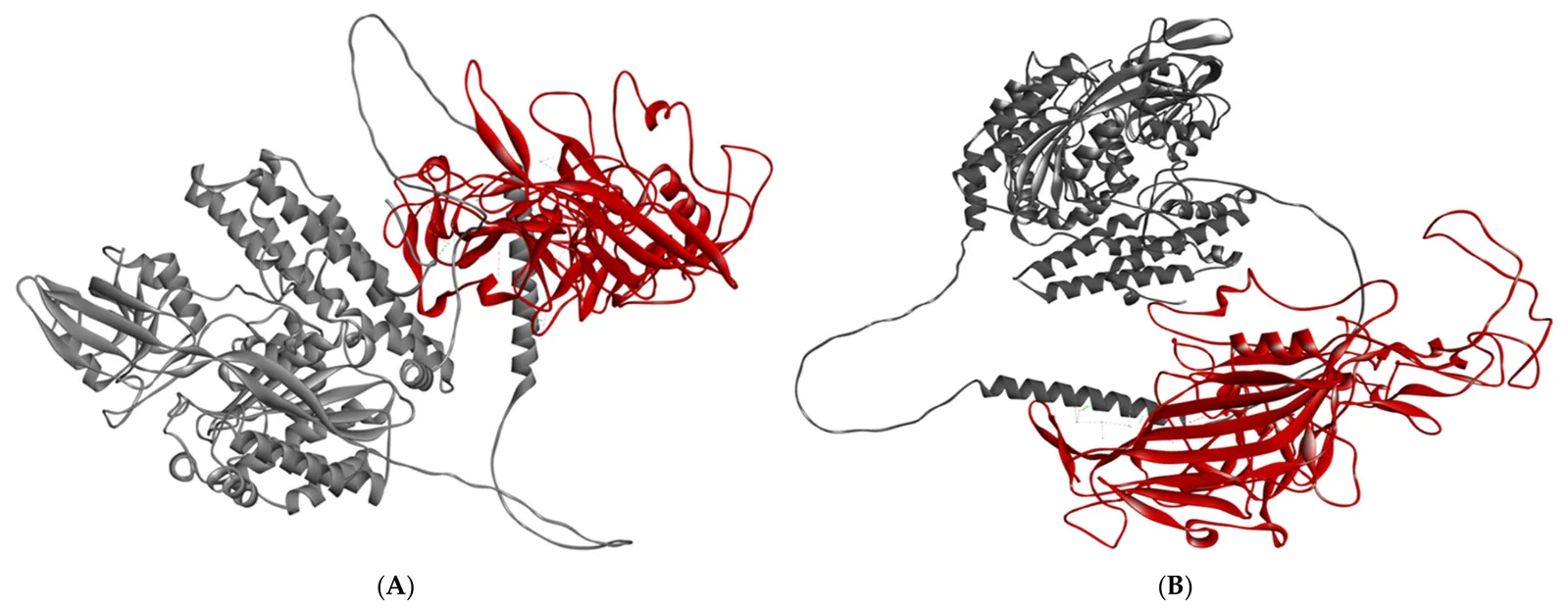
Docked structure of TfR1-VP2 complex of (**A**) Feline and (**B**) Canine showing receptor TfR1 (grey) and VP2 (red) interaction interface.

Comparative docking analysis revealed differences in binding characteristics between feline and canine systems (Figure 13A,B).

#### 3.6.1. Docking Scores

The docking analysis of TfR1 with VP2 revealed distinct interaction profiles between canine and feline hosts. The canine TfR1-VP2 complex demonstrated a slightly higher ZDOCK score (20.66) compared to the feline complex (19.74), suggesting better shape complementarity and initial docking fit. However, the feline TfR1-VP2 complex exhibited a significantly more favorable ZRANK score (−144.808) than the canine complex (−116.701), indicating enhanced binding stability and more energetically favorable interactions. This contrast suggested that while the canine complex may achieve a better geometric alignment, the feline complex forms a more stable and energetically optimized interaction. Overall, these findings indicate differences in the predicted receptor–virus interaction patterns between the two host systems.

#### 3.6.2. Physicochemical Properties of Docking Components

The physicochemical analysis of docking components are presented in Table 6. All proteins exhibited isoelectric points (pI) below the physiological pH of 7.4, resulting in an overall net negative charge under physiological conditions.

Among the receptors, feline TfR1 exhibited a more pronounced negative charge (−6.13) than canine TfR1 (−3.04), indicating a greater potential for electrostatic interactions with positively charged or polar regions of the ligand. The VP2 proteins from both hosts also showed negative net charges, with canine VP2 (−6.41) being slightly more negative than feline VP2 (−5.92). These variations in charge distribution suggested subtle differences in electrostatic complementarity between receptor and ligand in each host system. Overall, the higher negative charge observed in feline TfR1 may contribute to stronger or more stable binding interactions, supporting the docking results that indicated enhanced binding stability in the feline TfR1-VP2 complex.

#### 3.6.3. Interaction Analysis

Analysis of non-bonded contacts revealed several critical interactions that likely stabilize the local conformation and inter-residue packing. A summary of the major interactions identified in the feline and canine systems is presented in Table 7 and Table 8.

#### 3.6.4. Hydrogen Bonds

##### Feline Panleukopenia Virus

A strong conventional hydrogen bond was observed between LYS63(NZ) and GLU393(OE1) with a distance of 1.91 Å and a donor-H-acceptor angle of 106.2°, indicating a robust stabilizing contact. An even shorter hydrogen bond (1.81 Å) was found between ASN78(HD22) and ILE762(O) (angle 109.9°), suggested a very strong, possibly low-barrier hydrogen bond that may serve as an anchor at the interface. Additional conventional hydrogen bonds included GLY87(HN)-LEU478(O) (2.55 Å, 90.6°), TYR79(HN)-TRP763(O) (2.57 Å, 136.3°), and CYS70(HG)-ASP99(O) (2.97 Å, 116.5°). These interactions collectively contribute to local secondary structure stabilization.

##### Canine Parvovirus

The shortest interaction observed was a salt bridge/hydrogen bond hybrid between ARG64(HH12) and GLU104(OE2) at a distance of only 1.30 Å (donor-H-acceptor angle 113.3°), indicating an exceptionally strong, possibly low-barrier ionic-H-bond. A second, longer contact between ARG64(HH22) and the same GLU104(OE2) (2.77 Å, angle 99.8°) further reinforces this interaction. Additional conventional hydrogen bonds include ASN66(HN)-GLU193(OE1) (1.84 Å, 121.6°), ARG209(HH12)-ASN66(O) (2.30 Å, 149.0°), LYS63(HZ2)-ALA379(O) (2.56 Å, 130.8°), LYS61(HZ1)-ASP99(O) (2.73 Å, 114.5°), HIS70(HD1)-TYR71(O) (2.68 Å, 125.0°), and TYR165(HH)-LEU78(O) (2.83 Å, 122.4°). These hydrogen bonds collectively stabilize local secondary structure and loop regions.

#### 3.6.5. Electrostatic and Salt Bridge Interactions

##### Feline Panleukopenia Virus

A salt bridge between ARG81(NH1) and ASP766(OD2) was identified at a distance of 3.51 Å, consistent with a strong ionic interaction. An additional electrostatic contact was noted between LYS63(NZ) and GLU393(OE2) at 4.17 Å, which, together with the hydrogen bond from the same lysine to OE1, forms a dual-mode electrostatic/hydrogen-bonding motif with GLU393.

##### Canine Parvovirus

The ARG64-GLU104 pair acts as both a salt bridge and a hydrogen bond, with distances of 1.30 Å (HH12 to OE2) and 2.77 Å (HH22 to OE2). No other long-range electrostatic contacts were identified in the canine dataset.

#### 3.6.6. π-Mediated Interactions

##### Feline Panleukopenia Virus

A π–cation interaction was observed between ARG22(NH1) and the aromatic ring of TYR510 (distance 4.89 Å, angle θ = 35.7°). Additionally, a rare π-donor hydrogen bond was identified between ARG377(HE) and PHE68 (2.91 Å), and an amide-π stacked interaction between ASN508/GLN509 and PHE23 (5.23 Å).

##### Canine Parvovirus

Two amide-π stacked contacts involve PHE68 with the backbone amide groups of THR194/LEU195 (3.93 Å) and PRO205/THR206 (3.67 Å). The latter is notably short, suggesting a strong polar-π stabilization. Additionally, a π-σ interaction was identified between SER58 and TYR343 at 2.87 Å, representing an unusual but potentially significant contact for local packing.

#### 3.6.7. Hydrophobic Interactions

##### Feline Panleukopenia Virus

Several alkyl and π-alkyl contacts were identified, including PHE68-ARG377 (π-alkyl, 4.33 Å), TYR71-ALA97 (π-alkyl, 4.15 Å), PHE79-PRO356 (π-alkyl, 4.90 Å), PHE79-ILE484 (π-alkyl, 3.97 Å), and alkyl-alkyl interactions such as PRO356-LEU78 (4.57 Å) and ILE86-LEU478 (5.30 Å). These contribute to the hydrophobic core and tertiary packing.

##### Canine Parvovirus

Several π-alkyl contacts contribute to the hydrophobic core, including HIS70-ALA75 (3.84 Å), TYR524-LEU78 (3.90 Å), and TYR71-ILE204 (4.27 Å) and TYR71-PRO205 (4.59 Å). An alkyl-alkyl interaction between VAL59 and LEU98 (4.83 Å) was also observed. These contacts help maintain the tertiary fold.

## 4. Discussion

As the population of pet cats in India continues to increase steadily, the prevalence of emerging and re-emerging infectious diseases pose a growing threat to feline health. Chennai, a metropolitan city in southern India, supports a large and diverse companion animal population, with cats representing one of the most common household pets after dogs. Although core vaccines against FP, FHV, and FCV are widely available in India, comprehensive surveillance data on the prevalence of various viral diseases of cats and the antigenic relatedness between imported vaccine and circulating field strains is limited in India [12,15]. Therefore, the present study aimed to assess the FPV prevalence, diagnostic sampling, risk factors, genetic variations and nucleotide and amino acid substitutions of FPV strains, and genetic diversity between field and the vaccine strains of FPV among clinically suspected cats in Chennai, Tamil Nadu, southern India over the course of one year. In addition, molecular docking analyses were performed to explore the predicted interactions between transferrin receptor type 1 (TfR1) and VP2 protein.

In the present study, FPV DNA was detected in 46.11% (83/180) of clinically suspected cats presented in Chennai, Tamil Nadu, during the one-year study period. The findings of the present study were consistent with the observations of Abdel Baky [23], who reported 43% prevalence of FPV in Egypt for the period of one year from 2020 to 2021 through a clinico-epidemiological survey of cats, and Oguzoglu et al. [24] also reported 39% prevalence for FPV in domestic cats residing in Ankara, Turkey. However, Parthiban et al. [12] reported a prevalence of 25.3% FPV (17/67) with fecal samples collected from clinically suspected cats in Chennai, India from 2012 to 2013, whereas, Raja et al. [15] reported a lower prevalence rate of 7.14% FPV (3/42) by PCR in Chennai, Tamil Nadu, India for a four-months period from March to June 2022.

Fecal swabs were the most frequently collected clinical specimen in the present study, reflecting their routine use in cats suspected of feline panleukopenia. Previous studies have also reported the detection of FPV from fecal, rectal, oral, nasal, conjunctival and blood samples using molecular assays [8,25,26]. However, in the present dataset, PCR results were recorded at the animal level and not separately for each specimen type. Therefore, the relative diagnostic sensitivity or specificity of the different clinical specimens could not be determined. Further studies using paired specimens from the same animals and specimen-specific PCR results are required to identify the most suitable sample or combination of samples for FPV diagnosis.

The predominant clinical signs observed among FPV PCR-positive cats were diarrhoea, anorexia, and dehydration (87.95% each), followed by vomiting (86.75%). These findings are consistent with the known tropism of FPV for rapidly dividing intestinal and haematopoietic cells, and agree with the clinical observations reported by Kadam et al. [27]. Since all animals included in the study were clinically suspected cases, the clinical signs were interpreted descriptively rather than as independent risk factors.

Female cats had significantly higher odds of FPV PCR positivity than males after adjustment for other epidemiological factors. A similar predominance of FPV infection among female cats was reported in Bangladesh [28], whereas, studies from Egypt and the United Arab Emirates found no significant sex-associated difference [29,30]. Hormonal, reproductive, behavioural, or management-related differences between male and female cats may have contributed to the observed association, however, these factors were not specifically evaluated and require further investigation.

Vaccinated cats had lower adjusted odds of FPV PCR positivity than unvaccinated cats. This finding may reflect partial protection conferred by vaccination. However, detailed information on vaccine type, timing, number of doses, age at vaccination, booster status, and the interval between vaccination and sample collection were unavailable. Therefore, the observed association should be interpreted cautiously and cannot be considered a direct estimate of vaccine effectiveness.

Breed and age group were not significantly associated with FPV PCR positivity. Although variation in positivity was observed among individual breed and age categories, some groups contained few animals, particularly Maine Coon cats, and these differences should not be interpreted as evidence of breed susceptibility. Previous studies have similarly reported no significant association between breed and FPV infection [23,24,31,32]. Likewise, the absence of a significant age effect suggests that clinically susceptible cats may occur across different age groups, particularly when vaccination is incomplete or irregular [33].

Season was significantly associated with FPV PCR positivity. Compared with summer, higher adjusted odds were observed during the southwest and northeast monsoon periods, with the strongest association during the northeast monsoon. Increased humidity, moderate temperatures, congregation of animals, movement into shared shelters, and greater exposure to contaminated surroundings may have contributed to this seasonal pattern. However, the study covered only one year and included clinically suspected cats presented to selected veterinary facilities. Therefore, the observed seasonal association should be interpreted cautiously and confirmed through longer-term, multicentre surveillance. Differences from reports in other countries [34,35] may reflect regional variation in climate, animal management, vaccination practices, and study design. FPV is a highly contagious virus that is primarily transmitted via the fecal–oral route. Although FPV does not infect humans, human activities play a critical role in its mechanical spread among cat populations. People who handle infected cats or contaminated environments (litter boxes, bedding, food bowls, shoes, and clothing) can carry infectious viral particles on their hands, footwear, and fomites. In multi-cat households, shelters, and veterinary clinics, such human-mediated transmission significantly increases the risk of outbreaks. The virus is shed in large quantities in the faeces of infected cats, often before clinical signs appear, and can survive in the environment for up to one year [33]. When humans inadvertently transfer contaminated faeces or fomites to naive cats through shared spaces, the fecal–oral cycle is completed. Dense human–cat interactions in urban and peri-urban areas of India, where stray cats roam freely and pet cats are housed indoors, further amplify this risk. Therefore, effective control requires not only vaccination and isolation of sick cats but also rigorous hygiene practices by cat owners and veterinary staff to interrupt human-facilitated fecal–oral transmission.

The successful adaptation and propagation of the field FPV isolates in CRFK cells observed in the present study are consistent with previous reports describing the susceptibility of this cell line to feline panleukopenia virus infection. Characteristic cytopathic effects (CPEs), including cell rounding, shrinkage, aggregation, and detachment, were observed within 48–72 hpi and became more pronounced following successive blind passages, indicating successful viral adaptation to in vitro conditions and confirming the presence of infectious FPV. Of the 10 FPV-positive samples subjected to virus isolation, four isolates were successfully recovered and propagated in CRFK cells. Failure to isolate virus from the remaining samples may be attributed to low viral titres, reduced viral viability during sample collection, transport, or storage, the presence of inhibitory substances in clinical specimens, or differences in the stage of infection at the time of sampling. Similar observations have been reported previously, highlighting that successful virus isolation depends not only on PCR positivity but also on the presence of viable infectious virus particles.

In the present study, FPV-positive field samples shared 98.1–98.5% nucleotide identity with VP2 region of FPV vaccine strains and 98.8–100% nucleotide identity with strains of other countries. Similarly, Parthiban et al. [12] reported that FPV isolates from Tamil Nadu, India revealed 99% identity with FPV strains of other countries. Further, Mukhopadhyay et al. [14] observed the 99–100% nucleotide identity with FPV strains from the diverse geographical regions of India. Recently, Raja et al. [15] reported that the complete genome sequence of the FPV strain shared 99.75% nucleotide identity with FPV variant from Tamil Nadu, India.

Further, in the present study, the FPV-VP2 capsid protein gene revealed seven unique nucleotide variations comparing with vaccine strains. While comparing with other Indian FPV strains, six out of seven nucleotide variations were same except at position 3355 (C-G). Similarly, Parthiban et al. [12] reported that FPV isolates from Tamil Nadu, India revealed seven unique nucleotide variations when compared with FPV and CPV-2, CPV-2a and CPV-2c reference strains. Mukhopadhyay et al. [14] also identified six non-synonymous and 11 synonymous mutations in the CPV/FPV from different states of India; whereas, Hafenstein et al. [36] observed that minor genetic changes in major capsid protein of FPV could affect the antigenic structure and immune competence of the viruses.

In the present study, eight amino acid variations were identified among the FPV field strains, vaccine strain and FPV strains from India and other countries. Overall, the amino acid sequences of the field isolates showed a high degree of similarity to the vaccine strain currently used in India. Previous studies have reported that the point mutations within the VP2 protein may contribute to the antigenic differences between variants and target host differentiation [37]. However, the biological significance of the amino acid substitutions identified in the present study remains uncertain. Further studies incorporating antigenic characterization, neutralization assays, and functional analyses are required to determine whether these substitutions influence viral antigenicity, host range, or viral fitness.

Reports indicate that vaccine-induced or vaccine related infections may develop after vaccinating cats with FPV vaccines [38,39]. In the present study, 31.33% of FPV positive cats had a history of vaccination against FPV. However, detailed information regarding vaccine type, vaccination schedule, number of doses, booster status, age at vaccination, and the interval between vaccination and sample collection was unavailable. Therefore, the relationship between vaccination history and FPV infection could not be evaluated further, and no conclusions regarding vaccine effectiveness can be drawn from the present study.

Similar observations have been reported in other Asian countries, where circulating FPV strains continue to exhibit genetic diversity and evolutionary divergence from vaccine strains. A recent study from China demonstrated that most field FPV isolates clustered within a distinct phylogenetic subgroup, whereas vaccine strains were distributed in a separate subgroup, indicating genetic differentiation between circulating and vaccine-associated strains [40]. Furthermore, increasing genetic variation and the emergence of novel FPV variants have been reported, emphasizing the importance of continuous molecular epidemiological surveillance [40,41]. Similarly, evolutionary analyses of Chinese FPV strains have revealed ongoing genetic diversification and accumulation of mutations within the VP2 gene [42]. These findings are consistent with the present study, in which the field FPV strains exhibited nucleotide and amino acid differences compared with the vaccine reference strain and formed distinct phylogenetic groupings. Collectively, these observations suggest ongoing genetic diversification of circulating FPV strains in India and neighboring Asian countries and highlight the importance of continued molecular surveillance. However, the potential implications of these genetic differences for vaccine performance remain uncertain and require further investigation through detailed immunological and vaccine-efficacy studies.

Among the 13 sequenced FPV strains, one isolate clustered with both FPV and CPV strains within clade 3, indicating the close genetic relationship between these viruses. These findings support the need for comprehensive genomic analyses of both vaccine and circulating field strains in India. Previous studies have reported that commercial FPV vaccine strains clustered within the same FPV clade; whereas certain FPV strains from Thrissur, Kerala, India formed a different clade and were closely related to reference FPV strain from the USA [14]. Similarly, phylogenetic analysis have demonstrated divergent evolutionary patterns between FPV and CPV-2 field strains [43]. These findings support the continuous evolution and genetic diversification of FPV and related parvoviruses across different geographical regions.

The number of predicted peptides within the antigenic determinant region of VP2 differed between the field and vaccine strains analyzed in the present study, reflecting the genetic variations identified in the VP2 gene. Similar observations have been reported previously, where variations in predicted antigenic regions of NS1, NS2, VP1, and VP2 were associated with genetic differences among FPV strains [15]. However, these findings are based solely on computational prediction and should be interpreted with caution, as no experimental validation, such as antibody-binding or virus-neutralization assays, was performed. Therefore, the biological significance of the predicted epitope differences remains uncertain. Future studies incorporating immunological and functional assays are required to validate these predictions and determine their relevance to FPV antigenicity and vaccine-related investigations.

The molecular characterization performed in the present study was based on a 698 bp fragment of the VP2 gene. Although complete VP2 or whole-genome sequencing provides higher genomic resolution, the VP2 gene remains the most informative region for FPV molecular epidemiology because it contains the major antigenic determinants and host range-associated residues [15]. Previous whole-genome studies have demonstrated that FPV genomes are highly conserved, with relatively limited variation in non-VP2 genomic regions, whereas, most epidemiologically relevant substitutions have been identified within the VP2 gene. Therefore, sequencing this informative VP2 region enabled reliable phylogenetic comparisons and assessment of the genetic diversity of circulating strains.

Pairwise genetic distance analysis revealed that the field isolates were highly conserved among themselves, with nucleotide divergences ranging from 0.15 to 0.26%. This indicates that the currently circulating FPV strains in Chennai belong to a single, relatively homogeneous genetic lineage. In contrast, the genetic distance between field isolates and the vaccine reference strain was substantially higher (2.18–2.72%). This level of divergence, while moderate, is notable because the VP2 gene is the primary target of neutralizing antibodies. Similar levels of vaccine-field divergence have been reported for panleukopenia in other species and have been associated with reduced vaccine efficacy in some settings [7]. Although, the clinical significance of this 2–3% divergence remains to be determined, it underscores the need for regular vaccine matching studies in India.

Selection pressure analysis using the FEL method identified five codons (175, 178, 179, 190, and 232) under purifying selection, indicating strong functional constraints that may preserve critical structural or receptor-binding regions of the VP2 protein. Conversely, two codons (45 and 152) showed evidence of diversifying (positive) selection. The presence of diversifying selection at these sites may indicate ongoing adaptive evolution, potentially driven by host immune pressure or other evolutionary forces acting on circulating FPV strains. However, these findings should be interpreted with caution. A limitation of the present study is that the selection pressure analysis was performed using only 13 VP2 sequences derived from the sequenced FPV-positive samples. Although 83 samples were identified as FPV-positive by PCR, only a subset was subjected to sequencing for molecular characterization. Consequently, the relatively small sequence dataset may have reduced the statistical power and robustness of the FEL-based analysis. Therefore, the observed signals of purifying and diversifying selection should be considered preliminary until validated using larger sequence datasets representing broader geographic and temporal distributions.

Transferrin receptor type 1 (TfR1) serves as the primary cellular receptor for FPV and related carnivore parvoviruses. In the present study, molecular docking analysis identified differences in the predicted interaction patterns between feline and canine TfR1-VP2 complexes. Although the canine complex exhibited a slightly higher ZDOCK score, the feline complex demonstrated a more favourable ZRANK score, suggesting differences in the predicted stability of the docked complexes. Physicochemical analyses further revealed variations in net charge distribution between feline and canine TfR1 proteins, while interaction analyses identified differences in the predicted hydrogen bonds and salt-bridge interactions formed within the complexes. These findings provide a computational perspective on potential VP2–TfR1 interaction patterns in feline and canine systems. However, the biological significance of these predicted interactions remains uncertain because the analyses were based solely on computational modelling. Therefore, the results should not be interpreted as direct evidence of receptor affinity, host specificity, viral adaptation, or evolutionary relationships. Further validation through receptor-binding assays, site-directed mutagenesis, molecular dynamics simulations, and in vitro infection studies is required to determine the biological relevance of the predicted interactions.

Although the study provides clinically useful information on FPV detection, epidemiological associations, genetic variation, and virus host interaction, several limitations should be acknowledged. The present study included clinically suspected cats presented to the Madras Veterinary College Teaching Hospital and private veterinary clinics in and around Chennai, Tamil Nadu. Further, Chennai, being one of the largest metropolitan cities in India with a substantial pet and free-roaming cat population, serves as an important hub for companion animal healthcare and disease surveillance. However, because the study was hospital-based rather than a systematic population-based survey, the findings may not fully represent the epidemiological status of FPV across all regions of Tamil Nadu. Therefore, large-scale epidemiological studies involving broader geographical coverage are warranted to further validate the present findings. The broad age category of >3 years may have obscured differences among older cats, and future studies should record exact age and use more detailed adult and senior age categories. Detailed vaccination information, including vaccine type, number of doses, booster history, age at vaccination, and interval between vaccination and sampling, was not available, which limits interpretation of the observed association with vaccination status. Some breed and age categories included relatively few animals, reducing the precision of subgroup comparisons. In addition, only a partial region of the VP2 gene was sequenced, and the antigenic epitope and molecular docking findings were based on computational predictions without experimental or molecular dynamics validation. These limitations should be considered while interpreting the results, and future studies incorporating complete vaccination records, full-length VP2 or whole-genome sequencing, and experimental validation are warranted.

## 5. Conclusions

The present study provides a comprehensive investigation of FPV infection among clinically suspected cats presented to veterinary facilities in Chennai, Tamil Nadu. FPV DNA was detected in 46.11% (83/180) of the clinically suspected cats examined. Multivariable analysis showed higher odds of FPV PCR positivity in female cats and during the southwest and northeast monsoon seasons, whereas vaccinated cats exhibited lower odds of positivity. Age and breed were not significantly associated with FPV infection. Molecular characterization based on partial VP2 gene sequencing demonstrated that the circulating field strains shared 98.1–98.5% nucleotide identity with currently available vaccine strains, with seven unique nucleotide variations and eight amino acid substitutions in the VP2 protein. These findings highlight the genetic diversity of FPV strains circulating in southern India and emphasize the importance of continued molecular surveillance. Although differences were observed between field and vaccine strains, the potential implications for vaccine performance remain uncertain and require further investigation using detailed vaccination records and immunological studies. Virus isolation in CRFK cells confirmed the presence of infectious FPV in selected clinical samples, while phylogenetic and selection pressure analyses provided insights into the evolutionary characteristics of the circulating strains. In addition, computational analyses identified variations in predicted VP2 antigenic regions and virus–receptor interaction patterns. However, these findings are based solely on in silico predictions and should be interpreted with caution until validated through experimental studies. Overall, the present study contributes valuable epidemiological and molecular data on FPV circulating in India and underscores the importance of continuous surveillance of VP2 genetic variation to support disease monitoring, vaccine evaluation, and effective control strategies.

## Figures and Tables

**Table 1:** Multivariable logistic regression analysis of epidemiological factors associated with FPV PCR positivity among clinically suspected cats.

Factor	Category	Total (*n*)	PCR Positive, *n* (%)	Adjusted OR (95% CI)	Category *p*-Value	Overall *p*-Value
Sex	Male	94	33 (35.11)	1.00 (Reference)	-	0.004
	Female	86	50 (58.14)	2.65 (1.35–5.21)	0.005	
Age group	<3 months	39	15 (38.46)	1.00 (Reference)	-	0.328
	3–6 months	43	19 (44.19)	0.80 (0.29–2.20)	0.668	
	6–12 months	42	24 (57.14)	2.08 (0.73–5.88)	0.169	
	1–3 years	46	21 (45.65)	0.85 (0.31–2.32)	0.753	
	>3 years	10	4 (40.00)	0.69 (0.14–3.29)	0.643	
Breed	Domestic Shorthair	133	59 (44.36)	1.00 (Reference)	-	0.472
	Persian	20	8 (40.00)	0.68 (0.24–1.96)	0.478	
	Other breeds	27	16 (59.26)	1.56 (0.57–4.25)	0.387	
Vaccination status	Unvaccinated	123	60 (48.78)	1.00 (Reference)	-	0.037
	Vaccinated	57	23 (40.35)	0.41 (0.17–0.97)	0.043	
Season	Summer	28	6 (21.43)	1.00 (Reference)	-	<0.001
	Winter	24	8 (33.33)	1.41 (0.37–5.41)	0.613	
	Southwest monsoon	104	52 (50.00)	3.59 (1.26–10.20)	0.016	
	Northeast monsoon	24	17 (70.83)	17.11 (3.86–75.80)	<0.001	

Note: Odds ratios were adjusted simultaneously for sex, age group, breed group, vaccination status, and season. Reference categories were male, <3 months, Domestic Shorthair, unvaccinated, and summer. Other breeds included non-descriptive, Maine Coon, and Russian Blue cats. OR, odds ratio; CI, confidence interval.

**Table 2:** Details of samples subjected to sequencing with their accession numbers.

S. No	Date Collected	Source	Sample Type	Sample ID	Test	Sex/Age	Breed/Color/Weight	Result	% Identity	GenBank Accession Number
1	15 October 2023	Clinics	Fecal	13316	FPV	Female/2 months	DSH/Brown/1.4 kg	Positive	100.00%	PP783785
2	28 September 2023	Clinics	Fecal, Blood, Oral	512	FPV	Female/10 months	Russia/White	Positive	99.85%	PP783786
3	20 October 2023	Private Clinics	Fecal	K3	FPV	Male/4 months	DSH/Fawn/1 kg	Positive	99.69%	PP783787
4	1 November 2023	Clinics	Fecal	VIJAY	FPV	Male/ND	DSH/ND	Positive	99.84%	PP783788
5	3 January 2024	Clinics	Fecal	WEDNESDAY	FPV	Female/1 year	ND	Positive	99.84%	PP783789
6	13 October 2023	Clinics	Fecal	15104	FPV	Male/3 years	DSH/4.5 kg	Positive	100.00%	PP783790
7	13 December 2023	Clinics	Fecal	14754	FPV	Female/1 year	DSH/3.3 kg	Positive	100.00%	PP783791
8	4 May 2024	Clinics	Fecal	45845	FPV	Female/5 months	Persian	Positive	99.38%	PQ495725
9	4 May 2024	Clinics (MVC)	Fecal	46390	FPV	Male/1 year	DSH/4.5 kg	Positive	99.29%	PQ495726
10	9 May 2024	Pet Clinics	Blood	Sep-24	FPV	Female/1.5 years	DSH/4.5 kg	Positive	99.07%	PQ495727
11	6 February 2024	Postmortem	Organ	Jun-24	FPV	Female/1 year	DSH	Positive	99.38%	PQ495728
12	4 May 2024	Clinics	Fecal	46396	FPV	Female/45 days	DSH	Positive	99.38%	PQ495729
13	20 April 2024	Pet Clinics	Blood	1	FPV	Male/1 year	DSH	Positive	99.53%	PQ495730
14	ND	Vaccine (Commercial)	ND	ND	FPV	ND	ND	Positive	100.00%	PQ495731

Note: ND: Non-descriptive; FPV: Feline panleukopenia virus; DSH: Domestic Shorthair.

**Table 3:** Comparison of nucleotide variations in the FPV-positive samples with other Indian FPV strains.

Position	Vaccine Strains	FPV Field Strains from This Study	Other Indian Strain Reported Earlier
3005	T	C	C
3107	T	C	C
3278	T	C	C
3317	T	C	C
3342	T	C	C
3355	C	T	G
3403	C	T/C	T

**Table 4:** Comparison of amino acid variations for FPV samples with other Indian FPV strains.

Position	Vaccine Strain	FPV Field Strains from This Study	Other TN Strains Reported Earlier
96	I	T	T
130	F	S	S
151	H	C	C
159	F	S	S
162	R	G	G
170	I	V	V
187	F	S	S
200	V	A	A

**Table 5:** Detailed site-by-site results from the Fixed Effects Likelihood (FEL) analysis.

Codon	Alpha	Beta	Alpha = Beta	LRT	*p*-Value	Class
232	0.033	0.000	0.000	3.903	0.0482	Purifying
45	0.000	0.001	0.000	6.063	0.0138	Diversifying
152	0.000	0.055	0.041	4.059	0.0439	Diversifying
190	25.692	0.026	7.303	3.802	0.0512	Purifying
175	32.151	0.013	10.207	5.298	0.0214	Purifying
178	192.388	0.076	19.146	4.051	0.0441	Purifying
179	273.245	0.071	24.255	3.403	0.0651	Purifying

**Table 6:** Physicochemical properties of canine and feline TfR1 and VP2 proteins used in docking analysis.

Species	Component	Name	Role	Force Field	Charge Method	pH	pI	Net Charge
Canine	Receptor	TfR1	Receptor	CHARMm	Momany-Rone	7.4	6.95	−3.04
Canine	Ligand	VP2	Ligand	CHARMm	Momany-Rone	7.4	6.25	−6.41
Feline	Receptor	TfR1	Receptor	CHARMm	Momany-Rone	7.4	6.63	−6.13
Feline	Ligand	VP2	Ligand	CHARMm	Momany-Rone	7.4	6.41	−5.92

**Table 7:** Non-bonded Interactions in feline TfR1-VP2 Complex.

S. No.	Interaction Type	Residue 1 (Chain A)	Residue 2 (Chain A)	Distance (Å)	Angle (°)
1	Hydrogen Bond	LYS63 (NZ)	GLU393 (OE1)	1.91	106.2
2	Hydrogen Bond	ASN78 (HD22)	ILE762 (O)	1.81	109.9
3	Hydrogen Bond	GLY87 (HN)	LEU478 (O)	2.55	90.6
4	Hydrogen Bond	TYR79 (HN)	TRP763 (O)	2.57	136.3
5	Hydrogen Bond	CYS70 (HG)	ASP99 (O)	2.97	116.5
6	Electrostatic (Salt Bridge)	ARG81 (NH1)	ASP766 (OD2)	3.51	-
7	Electrostatic	LYS63 (NZ)	GLU393 (OE2)	4.17	-
8	π–Cation Interaction	ARG22 (NH1)	TYR510 (π system)	4.89	35.7

**Table 8:** Non-bonded Interactions in Canine TfR1-VP2 Complex.

S. No	Interaction Type	Residue 1 (Chain A)	Residue 2 (Chain A)	Distance (Å)	Angle (°)
1	Salt bridge/H-bond	ARG64 (HH12)	GLU104 (OE2)	1.30	113.3
2	Hydrogen bond	ASN66 (HN)	GLU193 (OE1)	1.84	121.6
3	Hydrogen bond	ARG209 (HH12)	ASN66 (O)	2.30	149.0
4	Hydrogen bond	LYS63 (HZ2)	ALA379 (O)	2.56	130.8
5	Hydrogen bond	LYS61 (HZ1)	ASP99 (O)	2.73	114.5
6	Hydrogen bond	HIS70 (HD1)	TYR71 (O)	2.68	125.0
7	Hydrogen bond	TYR165 (HH)	LEU78 (O)	2.83	122.4
8	Hydrogen bond	ARG64 (HH22)	GLU104 (OE2)	2.77	99.8
11	Amide–π interaction	THR194/LEU195	PHE68	3.93	-
12	Amide–π interaction	PRO205/THR206	PHE68	3.67	-
13	π–σ interaction	SER58	TYR343	2.87	-
14	π–alkyl interaction	HIS70	ALA75	3.84	-
15	π–alkyl interaction	TYR524	LEU78	3.90	-
16	Hydrophobic (alkyl)	VAL59	LEU98	4.83	-

## Data Availability

All data generated or analyzed during this study are included in this published article.

## Supplementary Materials

The following supporting information can be downloaded at: https://www.mdpi.com/article/10.3390/microorganisms14081642/s1, Table S1: Details of samples collected from clinically suspected domestic cats along with risk factors for FPV.

## Abbreviations

The following abbreviations are used in this manuscript:

aOR	Adjusted ODD’s Ratio
BLAST	Basic Local Alignment Search Tool
CI	Confidence Interval
CPV	Canine Panleukopenia
CPV-2	Canine Panleukopenia Type 2
CPE	Cytopathic Effects
CRFK	Crandell–Rees Feline Kidney
DMEM	Dulbecco’s Modified Eagle Medium
DNA	Deoxyribonucleic Acid
dN	Nonsynonymous Substitution Rate
dS	Synonymous Substitution Rate
ELISA	Enzyme-Linked Immunosorbent Assay
FBS	Fetal Bovine Serum
FCV	Feline Calicivirus
FCoV	Feline Coronavirus
FEL	Fixed Effects Likelihood
FeLV	Feline Leukemia Virus
FEV	Feline Enteric Virus
FHV	Feline Herpesvirus
FP	Feline Panleukopenia
FPV	Feline Panleukopenia
IEDB	Immune Epitope Database and Analysis Resource
MEGA	Molecular Evolutionary Genetics Analysis
MVCTH	Madras Veterinary College Teaching Hospital
NCBI	National Center for Biotechnology Information
NS1	Nonstructural Protein 1
NS2	Nonstructural Protein 2
OR	Odds Ratio
PBS	Phosphate-Buffered Saline
PCR	Polymerase Chain Reaction
pI	Isoelectric Point
RNA	Ribonucleic Acid
TfR1	Transferrin Receptor Type 1
VP1	Viral Protein 1
VP2	Viral Protein 2
