# Supplementary material for: Molecular Epidemiology and Genetic Characterization of Feline Panleukopenia Virus among Clinically Suspected Domestic Cats in Tamil Nadu, India
Source: Microorganisms. 2026 Jul 27;14(8):1642. doi: 10.3390/microorganisms14081642 (PMC13515424; doi:10.3390/microorganisms14081642)
Supplement: Supplementary file 1 [file microorganisms-14-01642-s001.zip › microorganisms-4416924-supplementary.pdf]

Supplementary Table S1. Details of samples collected from clinically suspected domestic cats along with risk factors for FPV

S. NO	DATE	SAMPLE TYPE	CASE NO	BREED	SEX	AGE	CLINICAL SIGNS	VACCINATIO NDETAILS	TEST PERFO RMED	RESULT	STATUS OF ANIMALS
1	15-09-2023	FECAL	133612	DSH	FEMALE	2 MONTH	VOMITING	VACCINATED	PCR	POSITIVE	LIVE
2	15-09-2023	FECAL	2023131053	DSH	MALE	5 MONTH	ANOREXIA<br>VOMITING	NOT VACCINATED	PCR	NEGATIVE	LIVE
3	15-09-2023	FECAL	001	DSH	MALE	4 MONTH	VOMITING	VACCINATED	PCR	NEGATIVE	LIVE
4	15-09-2023	FECAL	002	DSH	MALE	1.2 MONTH	VOMITING	VACCINATED	PCR	NEGATIVE	LIVE
5	15-09-2023	FECAL	003	DSH	FEMALE	4 MONTH	VOMITING,<br>DIARRHEA	NOT VACCINATED	PCR	POSITIVE	LIVE
6	19-09-2023	BLOOD	004	DSH	MALE	1YEAR	VOMITING	VACCINATED	PCR	NEGATIVE	LIVE
7	06-10-2023	FECAL	005	DSH	MALE	1YEAR	VOMITING	VACCINATED	PCR	NEGATIVE	LIVE
8	06-10-2023	FECAL	006	DSH	MALE	6 MONTH	VOMITING	NOT VACCINATED	PCR	POSITIVE	LIVE
9	06-10-2023	FECAL	007	DSH	FEMALE	8 MONTH	VOMITING	NOT VACCINATED	PCR	POSITIVE	LIVE
10	06-10-2023	FECAL	1	PERISON	FEMALE	5 MONTH	VOMITING	VACCINATED	PCR	NEGATIVE	LIVE
11	06-10-2023	FECAL	4	DSH	FEMALE	4 MONTH	ANOREXIA,<br>VOMITING,	VACCINATED	PCR	POSITIVE	LIVE
12	06-10-2023	FECAL	5	RUSSIAN/	FEMALE	1 YEAR	ANOREXIA,<br>VOMITING,	VACCINATED	PCR	POSITIVE	LIVE
13	10-10-2023	BLOOD	008	RUSSIAN/	FEMALE	1 YEAR	ANOREXIA,<br>VOMITING,	VACCINATED	PCR	NEGATIVE	LIVE
14	20-10-2023	FECAL	K1	DSH	MALE	4 MONTH	ANOREXIA,	VACCINATED	PCR	POSITIVE	LIVE

							VOMITING,				
15	20-10-2023	FECAL	K3	DSH	MALE	5MONTH	ANOREXIA, VOMITING,	VACCINATED	PCR	POSITIVE	DEATH
16	20-10-2023	FECAL	K4	DSH	MALE	4MONTH	ANOREXIA, VOMITING,	VACCINATED	PCR	POSITIVE	DEATH
17	20-10-2023	FECAL	K5	DSH	MALE	4MONTH	ANOREXIA, VOMITING,	VACCINATED	PCR	POSITIVE	DEATH
18	20-10-2023	FECAL	K6	RUSSIAN/	FEMALE	1YEAR	ANOREXIA, VOMITING,	VACCINATED	PCR	POSITIVE	DEATH
19	20-10-2023	FECAL	K7	RUSSIAN/	FEMALE	2YEAR	ANOREXIA, VOMITING,	VACCINATED	PCR	POSITIVE	LIVE
20	20-10-2023	FECAL	K8	PERISON	FEMALE	1 1/2 YEAR	ANOREXIA, VOMITING, DIARRHEA	VACCINATED	PCR	POSITIVE	LIVE
21	20-10-2023	FECAL	K9	PERISON	FEMALE	1YEAR	ANOREXIA, VOMITING, DIARRHEA	VACCINATED	PCR	POSITIVE	LIVE
22	20-10-2023	FECAL	K10	RUSSIAN/	MALE	1YEAR	ANOREXIA, VOMITING, DIARRHEA	VACCINATED	PCR	POSITIVE	LIVE
23	20-10-2023	FECAL	K11	PERISON	MALE	2YEAR	ANOREXIA, VOMITING, DIARRHEA	VACCINATED	PCR	NEGATIVE	LIVE
24	20-11-2023	FECAL	K12	DSH	MALE	1 1/2 YEAR	ANOREXIA, VOMITING, DIARRHEA	VACCINATED	PCR	POSITIVE	LIVE
25	20-11-2023	FECAL	K13	DSH	MALE	1YEAR	VOMITING	VACCINATED	PCR	POSITIVE	LIVE
26	01-11-2023	FECAL/ BLOOD	K14	DSH	MALE	1YEAR	VOMITING	VACCINATED	PCR	POSITIVE	LIVE

27	13-12-2023	FECAL	15104	DSH	MALE	3YRS	VOMITING, DIARRHEA	VACCINATED	PCR	NEGATIVE	LIVE
28	13-12-2023	FECAL	14754	RUSSIN	FEMALE	1YRS	DIARRHEA	VACCINATED	PCR	NEGATIVE	LIVE
29	18-12-2023	FECAL	zara	RUSSIN	FEMALE	4MONTH	VOMITING, DIARRHEA	NOT VACCINATED	PCR	POSITIVE	LIVE
30	29-12-2023	FECAL	mimo	ND	MALE	1YEAR	VOMITING, DIARRHEA	NOT VACCINATED	PCR	NEGATIVE	LIVE
31	03-01-2024	FECAL/ ORAL	WEDNESDAY	RUSSIN	FEMALE	1YEAR	VOMITING, DIARRHEA	NOT VACCINATED	PCR	POSITIVE	LIVE
32	08-01-2024	FECAL	21858	ND	MALE	1YRS	ANOREXIA	NOT VACCINATED	PCR	POSITIVE	LIVE
33	11-01-2024	FECAL	22413	DSH	MALE	5MONTH	VOMITING, DIARRHEA	NOT VACCINATED	PCR	POSITIVE	LIVE
34	12-01-2024	FECAL/CONJUCTIVE SWAB	22508	RUSSIN	FEMALE	4MONTH/	INAPPERTENT	VACCINATED	PCR	NEGATIVE	LIVE
35	12-01-2024	FECAL	22628	DSH	FEMALE	15DAYS	DIARRHEA	VACCINATED	PCR	NEGATIVE	LIVE
36	12-01-2024	FECAL/CONJUCTIVE SWAB	21944	DSH	FEMALE	3MONTHS	VOMITING, DIARRHEA, BLINDNESS	VACCINATED	PCR	NEGATIVE	LIVE
37	12-01-2024	FECAL/CONJUCTIVE SWAB	27833	DSH	MALE	3MONTH	VOMITING, DIARRHEA	NOT VACCINATED	PCR	NEGATIVE	LIVE
38	18-01-2024	FECAL/CONJUCTIVE SWAB	23074	DSH	FEMALE	3MONTH	VOMITING, ANOREXIA	VACCINATED	PCR	POSITIVE	LIVE
39	22-01-2024	FECAL	24620	DSH	MALE	4MONTH	ANOREXIA,	VACCINATED	PCR	NEGATIVE	LIVE
40	01-02-2024	FECAL	MRS.AMUDHA	ND	FEMALE	3MONTHS	ANOREXIA,	NOT VACCINATED	PCR	NEGATIVE	LIVE
41	13-01-2024	FECAL	22418	PERISON	FEMALE	2.5YRS	VOMITING, DIARRHEA	VACCINATED	PCR	NEGATIVE	LIVE

42	13-01-2024	FECAL	3207	DSH	MALE	5MONTHS	VOMITING,<br>DIARRHEA	VACCINATED	PCR	NEGATIVE	LIVE
43	13-01-2024	FECAL	22738	DSH	MALE	8MONTH	VOMITING,<br>DIARRHEA	VACCINATED	PCR	NEGATIVE	LIVE
44	06-02-2024	ORGANS	22739	DSH	FEMALE	1YRS	PLEURAL EFFUSION	NOT VACCINATED	PCR	POSITIVE	DEATH
45	10-02-2024	FECAL/CONJUCT IVE SWAB	28794	DSH	MALE	4MONTH	VOMITING	VACCINATED	PCR	NEGATIVE	LIVE
46	10-02-2024	FECAL/CONJUCT IVE SWAB	28822	DSH	MALE	6MONTH	ANOREXIA,	NOT VACCINATED	PCR	NEGATIVE	LIVE
47	12-02-2024	FECAL/ BLOOD	28828	DSH	MALE	1YRS	ANOREXIA,	VACCINATED	PCR	POSITIVE	DEATH
48	13-02-2024	BLOOD	29310	DSH	FEMALE	6MONTH	ANOREXIA,	NOT VACCINATED	PCR	POSITIVE	LIVE
49	13-02-2024	FECAL	29425	PERISON	MALE	4MONTH	VOMITING	VACCINATED	PCR	NEGATIVE	LIVE
50	13-02-2024	FECAL/CONJUCT IVE SWAB/ORAL	29309	DSH	FEAMLE	4MONTH	ANOREXIA,	NOT VACCINATED	PCR	NEGATIVE	LIVE
51	19-02-2024	FECAL/CONJUCT IVE SWAB/ORAL	29915	DSH	MALE	8MONTH	ANOREXIA,	NOT VACCINATED	PCR	POSITIVE	LIVE
52	23-02-2024	BLOOD	ROSEY CAT	DSH	FEMALE	8MONTH	VOMITING,<br>DIARRHEA	NOT VACCINATED	PCR	NEGATIVE	LIVE
53	26-02-2024	FECAL/CONJUCT IVE SWAB/ORAL	SHOBA CAT	DSH	FEMALE	9MONTH	ANOREXIA,	NOT VACCINATED	PCR	NEGATIVE	LIVE
54	27-02-2024	FECAL/CONJUCT IVE SWAB/ORAL	34409	DSH	MALE	5MONTH	ANOREXIA,	NOT VACCINATED	PCR	NEGATIVE	LIVE
55	07-03-2024	FECAL/CONJUCT IVE SWAB	34410	DSH	MALE	5MONTH	PLEURAL EFFUSION	VACCINATED	PCR	NEGATIVE	LIVE
56	08-03-2024	FECAL/CONJUCT IVE SWAB	34558	RUSSIAN	FEMALE	1YEAR	PLEURAL EFFUSION	NOT VACCINATED	PCR	NEGATIVE	LIVE
57	11-03-2024	FECAL/CONJUCT IVE SWAB	35394	RUSSIAN	FEMALE	1YEAR	PLEURAL EFFUSION	NOT VACCINATED	PCR	NEGATIVE	LIVE
58	18-03-2024	DIGESTIVE FLUID	1	PERISON	FEMALE	9MONTH	PLEURAL EFFUSION	NOT VACCINATED	PCR	NEGATIVE	LIVE

59	19-03-2024	FECAL/CONJUCTIVE SWAB	37250	DSH	MALE	4MONTH	ANOREXIA, VOMITING, NEROLOGICAL SIGN	NOT VACCINATED	PCR	NEGATIVE	LIVE
60	20-03-2024	FECAL/ CONJUCTIVE SWAB/ DIGESTIVE FLUID	36315	DSH	FEMALE	8MONTHS	ANOREXIA, VOMITING, NEROLOGICAL SIGN	NOT VACCINATED	PCR	POSITIVE	LIVE
61	13-04-2024	FECAL/ CONJUCTIVE SWAB	41957	DSH	MALE	4MONTH	VOMITING	NOT VACCINATED	PCR	NEGATIVE	LIVE
62	13-04-2024	FECAL/ CONJUCTIVE SWAB	41976	DSH	MALE	5MONTH	VOMITING	NOT VACCINATED	PCR	NEGATIVE	LIVE
63	13-04-2024	FECAL/ CONJUCTIVE SWAB	41993	DSH	MALE	8MONTH	VOMITING	NOT VACCINATED	PCR	NEGATIVE	LIVE
64	13-04-2024	FECAL/ CONJUCTIVE SWAB	41943	DSH	MALE	1YEAR	VOMITING	NOT VACCINATED	PCR	NEGATIVE	LIVE
65	13-04-2024	FECAL/ CONJUCTIVE SWAB	41974	PERISON	MALE	3MONTH		VACCINATED	PCR	NEGATIVE	LIVE
66	13-04-2024	FECAL/ CONJUCTIVE SWAB	41638	ND	MALE	1.5YRS		NOT VACCINATED	PCR	NEGATIVE	LIVE
67	13-04-2024	FECAL/ CONJUCTIVE SWAB	41881	DSH	FEMALE	3YRS	VOMITING, DIARRHEA	NOT VACCINATED	PCR	POSITIVE	LIVE
68	13-04-2024	FECAL/ CONJUCTIVE SWAB	41903	DSH	MALE	8MONTH	VOMITING	NOT VACCINATED	PCR	POSITIVE	LIVE
69	13-04-2024	FECAL/ CONJUCTIVE SWAB	42150	DSH	MALE	8MONTH	VOMITING	NOT VACCINATED	PCR	NEGATIVE	LIVE

70	13-04-2024	FECAL/ CONJUCTIVE SWAB	41033	DSH	MALE	2MONTH	VOMITING	VACCINATED	PCR	NEGATIVE	LIVE
71	20-04-2024	FECAL	3	DSH	MALE	1MONTH	VOMITING, DIARRHEA	NOT VACCINATED	PCR	NEGATIVE	LIVE
72	20-04-2024	FECAL	4	DSH	MALE	1MONTH	VOMITING	NOT VACCINATED	PCR	NEGATIVE	LIVE
73	20-04-2024	FECAL	5	DSH	MALE	1MONTH	VOMITING	NOT VACCINATED	PCR	NEGATIVE	LIVE
74	20-04-2024	FECAL	6	DSH	FEMALE	1MONTH	VOMITING	VACCINATED	PCR	NEGATIVE	LIVE
75	04-05-2024	FECAL/ CONJUCTIVE SWAB	45845	PERISON	FEMALE	5MONTHS/	VOMITING, DIARRHEA	NOT VACCINATED	PCR	NEGATIVE	LIVE
76	04-05-2024	FECAL/ CONJUCTIVE SWAB	46411	DSH	MALE	3MONTH	VOMITING, DIARRHEA	NOT VACCINATED	PCR	NEGATIVE	LIVE
77	04-05-2024	FECAL/ CONJUCTIVE SWAB	46396	DSH	MALE	45DAYS	VOMITING, DIARRHEA	NOT VACCINATED	PCR	POSITIVE	LIVE
78	04-05-2024	FECAL/ CONJUCTIVE SWAB	46390	DSH	MALE	1MONTH/	VOMITING, DIARRHEA	NOT VACCINATED	PCR	POSITIVE	LIVE
79	04-05-2024	FECAL/ CONJUCTIVE SWAB	46409	DSH	MALE	1YEAR		NOT VACCINATED	PCR	NEGATIVE	LIVE
80	10-05-2024	FECAL/ CONJUCTIVE SWAB	46363	DSH	FEMALE	2 1/2 YEAR	DIARRHEA	VACCINATED	PCR	POSITIVE	LIVE
81	16-05-2024	FECAL/ CONJUCTIVE SWAB	47638	DSH	FEMALE	6 MONTHS	VOMITING	VACCINATED	PCR	NEGATIVE	LIVE
82	21-05-2024	FECAL/ CONJUCTIVE SWAB	40689	DSH	FEMALE	1.5YEAR	VOMITING, ANOREXIA	VACCINATED	PCR	NEGATIVE	LIVE
83	19-06-2024	FECAL SWAB	63075	DSH	MALE	8MONTHS	VOMITING	NON-VACCINATED	PCR	POSITIVE	LIVE

84	24-06-2024	FECAL SWAB	60643	DSH	MALE	6MONTH	VOMITING	NON-VACCINATED	PCR	POSITIVE	LIVE
85	04-07-2024	FECAL SWAB	62395	DSH	MALE	5MONTH	VOMITING	NON-VACCINATED	PCR	POSITIVE	LIVE
86	22-07-2024	FECAL	60643	DSH	MALE	4MONTH	VOMITING, ANOREXIA, DEPRESSED	NON-VACCINATED	PCR	NEGATIVE	LIVE
87	22-07-2024	FECAL	106549	DSH	MALE	3MONTH	VOMITING, ANOREXIA, DEPRESSED	NON-VACCINATED	PCR	NEGATIVE	LIVE
88	23-07-2024	FECAL	63327	DSH	MALE	4MONTH	VOMITING, ANOREXIA, DEPRESSED	NON-VACCINATED	PCR	NEGATIVE	LIVE
89	23-07-2024	FECAL	60218	DSH	MALE	2MONTH	VOMITING, ANOREXIA, DEPRESSED	NON-VACCINATED	PCR	NEGATIVE	LIVE
90	23-07-2024	FECAL	63385	DSH	MALE	5MONTH	VOMITING, ANOREXIA, DEPRESSED	NON-VACCINATED	PCR	NEGATIVE	LIVE
91	23-07-2024	FECAL	63540	DSH	MALE	4MONTH	VOMITING, ANOREXIA, DEPRESSED	NON-VACCINATED	PCR	NEGATIVE	LIVE
92	23-07-2024	FECAL	48813	DSH	MALE	6MONTH	VOMITING, ANOREXIA, DEPRESSED	NON-VACCINATED	PCR	NEGATIVE	LIVE
93	23-07-2024	FECAL	63557	DSH	MALE	4MONTH	VOMITING, ANOREXIA, DEPRESSED	NON-VACCINATED	PCR	NEGATIVE	LIVE

94	24-07-2024	FECAL	63328	DSH	MALE	3MONTH	VOMITING,<br>ANOREXIA,<br>DEPRESSED	NON-VACCINATED	PCR	NEGATIVE	LIVE
95	24-07-2024	FECAL	63368	DSH	MALE	4MONTH	VOMITING,<br>ANOREXIA,<br>DEPRESSED	NON-VACCINATED	PCR	NEGATIVE	LIVE
96	24-07-2024	FECAL	62509	DSH	MALE	4MONTH	VOMITING,<br>ANOREXIA,<br>DEPRESSED	NON-VACCINATED	PCR	NEGATIVE	LIVE
97	24-07-2024	FECAL	62402	DSH	MALE	4MONTH	VOMITING,<br>ANOREXIA,<br>DEPRESSED	NON-VACCINATED	PCR	NEGATIVE	LIVE
98	24-07-2024	FECAL	63598	DSH	FEAMLE	1.2YRS	VOMITING,<br>ANOREXIA,<br>DEPRESSED	NOT	PCR	POSITIVE	LIVE
99	24-07-2024	FECAL	62706	DSH	FEAMLE	1YRS3MONTH	VOMITING,<br>DIARRHEA	Vaccinated	PCR	NEGATIVE	LIVE
100	24-07-2024	FECAL	62489	DSH	FEAMLE	1YRS	VOMITING,<br>DIARRHEA	NOT	PCR	POSITIVE	LIVE
101	24-07-2024	FECAL/ CONJUCTIVE SWAB/ORAL	63743	ND	MALE	2YRS	VOMITING,<br>DIARRHEA	NOT	PCR	NEGATIVE	LIVE
102	24-07-2024	FECAL/ CONJUCTIVE SWAB/ORAL	63778	DSH	MALE	3YRS	VOMITING,<br>DIARRHEA	Vaccinated	PCR	NEGATIVE	LIVE
103	24-07-2024	FECAL/ CONJUCTIVE SWAB/ORAL	63682	PERISON	FEMALE	3MONTH	VOMITING,<br>DIARRHEA	NOT	PCR	POSITIVE	LIVE

104	25-07-2024	FECAL	63835	ND	MALE	1YRS	VOMITING, ANOREXIA, DEPRESSED	Vaccinated	PCR	NEGATIVE	LIVE
105	25-07-2024	FECAL	64749	MAINE LOON	MALE	8MONTH	ANOREXIA,	Vaccinated	PCR	POSITIVE	LIVE
106	25-07-2024	FECAL	64956	DSH	FEMALE	3MONTH	ANOREXIA,	Vaccinated	PCR	NEGATIVE	LIVE
107	25-07-2024	FECAL	64923	DSH	MALE	1YRS	VOMITING, DIARRHEA	Vaccinated	PCR	NEGATIVE	LIVE
108	25-07-2024	FECAL	64408	ND	MALE	1YRS	VOMITING, DIARRHEA	NOT	PCR	POSITIVE	LIVE
109	30-07-2024	FECAL	63943	DSH	MALE	6MONTH	DIARRHEA	NOT	PCR	NEGATIVE	LIVE
110	30-07-2024	FECAL	64968	ND	FEMALE	5MONTH	VOMITING, DIARRHEA	NOT	PCR	POSITIVE	LIVE
111	30-07-2024	FECAL	68449	ND	FEMALE	7MONTH	DIARRHEA	NOT	PCR	NEGATIVE	LIVE
112	30-07-2024	FECAL	66271	ND	FEMALE	6MONTH	ANOREXIA, VOMITING, DIARRHEA	Vaccinated	PCR	POSITIVE	LIVE
113	30-07-2024	FECAL	66308	ND	FEMALE	6MONTH	ANOREXIA, VOMITING, DIARRHEA	Vaccinated	PCR	POSITIVE	LIVE
114	30-07-2024	FECAL	66312	DSH	FEMALE	9MONTH	ANOREXIA, VOMITING, DIARRHEA	Vaccinated	PCR	POSITIVE	LIVE
115	06-08-2024	FECAL	66521	DSH	MALE	1YRS	ANOREXIA, VOMITING, DIARRHEA	NOT	PCR	NEGATIVE	LIVE
116	06-08-2024	FECAL	66272	ND	MALE	4YRS	ANOREXIA, VOMITING, DIARRHEA	Vaccinated	PCR	NEGATIVE	LIVE

117	06-08-2024	FECAL	66139	DSH	MALE	6MONTH	ANOREXIA, VOMITING, DIARRHEA	NOT	PCR	POSITIVE	LIVE
118	06-08-2024	FECAL	66625	DSH	FEMALE	2YRS	ANOREXIA, VOMITING, DIARRHEA	NOT	PCR	NEGATIVE	LIVE
119	06-08-2024	FECAL	64966	DSH	FEMALE	1YRS	ANOREXIA, VOMITING, DIARRHEA	Vaccinated	PCR	NEGATIVE	LIVE
120	07-08-2024	FECAL	66410	ND	MALE	9MONTH	ANOREXIA, VOMITING, DIARRHEA	NOT	PCR	POSITIVE	LIVE
121	07-08-2024	FECAL	63466	DSH	FEMALE	3MONTH	ANOREXIA, VOMITING, DIARRHEA	NOT	PCR	NEGATIVE	LIVE
122	07-08-2024	FECAL	66836	DSH	FEMALE	3MONTH	ANOREXIA, VOMITING, DIARRHEA	Vaccinated	PCR	NEGATIVE	LIVE
123	07-08-2024	FECAL	66793	DSH	FEMALE	7MONTH	ANOREXIA, VOMITING, DIARRHEA	NON-VACCINATED	PCR	POSITIVE	LIVE
124	08-08-2024	FECAL	67811	DSH	FEMALE	4MONTH	ANOREXIA, VOMITING, DIARRHEA	NON-VACCINATED	PCR	NEGATIVE	LIVE
125	08-08-2024	FECAL	67890	DSH	MALE	11MONTH	ANOREXIA, VOMITING, DIARRHEA	NON-VACCINATED	PCR	POSITIVE	LIVE

126	12-08-2024	FECAL	66143	DSH	FEMALE	4MONTH	ANOREXIA,<br>VOMITING,<br>DIARRHEA	NON-VACCINATED	PCR	POSITIVE	LIVE
127	12-08-2024	FECAL	67916	DSH	FEMALE	3 1/2 MONTH	VOMITING,<br>DIARRHEA,	NON-VACCINATED	PCR	POSITIVE	LIVE
128	12-08-2024	FECAL	67913	DSH	FEMALE	1.5YRS	VOMITING,<br>DIARRHEA,	NON-VACCINATED	PCR	POSITIVE	LIVE
129	12-08-2024	FECAL	70175	DSH	FEMALE	9MONTH	VOMITING,<br>DIARRHEA	NON-VACCINATED	PCR	POSITIVE	LIVE
130	12-08-2024	FECAL	71204	DSH	FEMALE	2YRS	VOMITING,<br>DIARRHEA	NON-VACCINATED	PCR	POSITIVE	LIVE
131	12-08-2024	FECAL	70696	DSH	MALE	11MONTH	VOMITING,<br>DIARRHEA	VACCINATED	PCR	NEGATIVE	LIVE
132	12-08-2024	FECAL	71053	DSH	FEMALE	1YRS	VOMITING,<br>DIARRHEA	NON-VACCINATED	PCR	POSITIVE	LIVE
133	28-08-2024	FECAL	70958	DSH	FEMALE	1MONTH	DEHYDRATED<br>, DEPRESSED,<br>ANOREXIA	Vaccinated	PCR	NEGATIVE	LIVE
134	28-08-2024	FECAL	70766	DSH	FEMALE	9MONTH	VOMITING,<br>DIARRHEA	NON-VACCINATED	PCR	POSITIVE	LIVE
135	28-08-2024	FECAL	70383	PERISON	MALE	8MONTH	DEHYDRATED,<br>DEPRESSED,<br>ANOREXIA	NON-VACCINATED	PCR	POSITIVE	LIVE
136	28-08-2024	FECAL	70881	PERISON	MALE	2MONTH	VOMITING,<br>DIARRHEA	NON-VACCINATED	PCR	POSITIVE	LIVE

137	28-08-2024	FECAL	70848	DSH	MALE	1YRS	DEHYDRATED, DEPRESSED, ANOREXIA	NON-VACCINATED	PCR	NEGATIVE	LIVE
138	28-08-2024	FECAL	58	DSH	FEMALE	4MONTH	VOMITING, DIARRHEA	NON-VACCINATED	PCR	POSITIVE	LIVE
139	29-08-2024	FECAL	71451	DSH	FEMALE	1YRS	VOMITING, DIARRHEA	NON-VACCINATED	PCR	POSITIVE	LIVE
140	29-08-2024	FECAL	71475	PERISON	MALE	5MONTH	VOMITING, DIARRHEA	NON-VACCINATED	PCR	NEGATIVE	LIVE
141	29-08-2024	FECAL	69387	ND	FEMALE	1YRS	VOMITING, DIARRHEA	NON-VACCINATED	PCR	POSITIVE	LIVE
142	29-08-2024	FECAL	71481	ND	FEMALE	4MONTH	DEHYDRATED, DEPRESSED, ANOREXIA	NON-VACCINATED	PCR	POSITIVE	LIVE
143	30-08-2024	FECAL	71552	PERISON	MALE	6MONTH	VOMITING, DIARRHEA	NON-VACCINATED	PCR	POSITIVE	LIVE
144	30-08-2024	FECAL	79551	DSH	FEMALE	9MONTH	VOMITING, DIARRHEA	NON-VACCINATED	PCR	POSITIVE	LIVE
145	30-08-2024	FECAL	71556	DSH	FEMALE	6MONTH	VOMITING, DIARRHEA	NON-VACCINATED	PCR	POSITIVE	LIVE
146	30-08-2024	FECAL/ CONJUCTIVE SWAB/ORAL/ NASAL SWAB	71558	DSH	FEMALE	3YRS	VOMITING, DIARRHEA	NON-VACCINATED	PCR	POSITIVE	LIVE
147	30-08-2024	FECAL	71565	DSH	FEMALE	2YRS	VOMITING, DIARRHEA	NON-VACCINATED	PCR	POSITIVE	LIVE

148	30-08-2024	FECAL /CONJUCTIVE SWAB/ ORAL/NASAL SWAB	72018	DSH	MALE	1YRS	VOMITING,<br>DIARRHEA	NON-VACCINATED	PCR	NEGATIVE	LIVE
149	30-08-2024	FECAL/ CONJUCTIVE SWAB/ORAL	71384	DSH	MALE	1 1/2 YRS	VOMITING,<br>DIARRHEA	Vaccinated	PCR	POSITIVE	LIVE
150	30-08-2024	FECAL	72057	DSH	FEMALE	8MONTH	ANOREXIA,<br>VOMITING,	NON-VACCINATED	PCR	POSITIVE	LIVE
151	30-08-2024	FECAL	72064	DSH	FEMALE	7MONTH	ANOREXIA,<br>VOMITING,	NON-VACCINATED	PCR	POSITIVE	LIVE
152	02-09-2024	FECAL	71704	DSH	FEMALE	3MONTH	ANOREXIA,<br>VOMITING,	NON-VACCINATED	PCR	POSITIVE	LIVE
153	02-09-2024	FECAL	71705	DSH	FEMALE	3MONTH	ANOREXIA,<br>VOMITING,	NON-VACCINATED	PCR	POSITIVE	LIVE
154	02-09-2024	FECAL	71703	DSH	FEMALE	3MONTH	ANOREXIA,<br>VOMITING,	NON-VACCINATED	PCR	POSITIVE	LIVE
155	02-09-2024	FECAL	71710	DSH	FEMALE	3MONTH	ANOREXIA,<br>VOMITING	NON-VACCINATED	PCR	POSITIVE	LIVE
156	03-09-2024	FECAL	70785	PERISON	FEMALE	1YRS	ANOREXIA,<br>VOMITING	NON-VACCINATED	PCR	POSITIVE	LIVE
157	03-09-2024	FECAL	72444	DSH	FEMALE	5MONTH	ANOREXIA,<br>VOMITING	NON-VACCINATED	PCR	POSITIVE	LIVE
158	03-09-2024	FECAL	72263	PERISON	FEMALE	1YRS	VOMITING,<br>DIARRHEA	NON-VACCINATED	PCR	NEGATIVE	LIVE
159	03-09-2024	FECAL	72493	DSH	MALE	2MONTH	ANOREXIA	NON-VACCINATED	PCR	POSITIVE	LIVE

160	04-09-2024	FECAL	71552	DSH	MALE	5MONTH	ANOREXIA,<br>VOMITING,	NON-VACCINATED	PCR	NEGATIVE	LIVE
161	04-09-2024	FECAL	72565	DSH	FEMALE	6MONTH	ANOREXIA	NON-VACCINATED	PCR	POSITIVE	LIVE
162	04-09-2024	FECAL	72561	DSH	FEMALE	8MONTH	ANOREXIA	NON-VACCINATED	PCR	POSITIVE	LIVE
163	04-09-2024	FECAL	72582	DSH	MALE	8MONTH	ANOREXIA	NON-VACCINATED	PCR	NEGATIVE	LIVE
164	04-09-2024	FECAL	72496	DSH	FEMALE	2YRS	ANOREXIA	NON-VACCINATED	PCR	NEGATIVE	LIVE
165	04-09-2024	FECAL	72268	DSH	FEMALE	3YRS	VOMITING<br>, DIARRHEA	NON-VACCINATED	PCR	NEGATIVE	LIVE
166	04-09-2024	FECAL	72579	DSH	FEMALE	3MONTH	ANOREXIA	NON-VACCINATED	PCR	NEGATIVE	LIVE
167	05-09-2024	FECAL	72669	DSH	FEMALE	6MONTH	ANOREXIA	NON-VACCINATED	PCR	NEGATIVE	LIVE
168	05-09-2024	FECAL	72165	DSH	FEMALE	2.5MONTH	DEHYDRATED<br>DEPRESSED,<br>ANOREXIA	NON-VACCINATED	PCR	POSITIVE	LIVE
169	05-09-2024	FECAL	72371	DSH	FEMALE	2YRS	DEHYDRATED	NON-VACCINATED	PCR	NEGATIVE	LIVE
170	05-09-2024	FECAL	71785	PERISON	MALE	1YRS	DEHYDRATED,	NON-VACCINATED	PCR	NEGATIVE	LIVE
171	05-09-2024	FECAL	72729	DSH	MALE	1MONTH	DEHYDRATED,	NON-VACCINATED	PCR	POSITIVE	LIVE

172	05-09-2024	FECAL	72746	DSH	FEMALE	3MONTH	DEHYDRATED,	NON-VACCINATED	PCR	POSITIVE	LIVE
173	05-09-2024	FECAL	72626	DSH	FEMALE	2YRS	DEHYDRATED	NON-VACCINATED	PCR	NEGATIVE	LIVE
174	05-09-2024	FECAL	72781	DSH	FEMALE	2YRS	DEHYDRATED	NON-VACCINATED	PCR	NEGATIVE	LIVE
175	06-09-2024	FECAL	73110	DSH	MALE	5MONTH	ANOREXIA	NON-VACCINATED	PCR	NEGATIVE	LIVE
176	06-09-2024	FECAL	73116	PERISON	FEMALE	2.5MONTH	ANOREXIA<br>VOMITING	NON-VACCINATED	PCR	POSITIVE	LIVE
177	06-09-2024	FECAL	73138	PERISON	MALE	6YRS	ANOREXIA	NON-VACCINATED	PCR	NEGATIVE	LIVE
178	06-09-2024	FECAL	71538	DSH	FEMALE	3MONTH	DEHYDRATED<br>ANOREXIA	NON-VACCINATED	PCR	NEGATIVE	LIVE
179	09-09-2024	FECAL	73043	PERISON	MALE	3YRS	VOMITING<br>DIARRHEA	NON-VACCINATED	PCR	NEGATIVE	LIVE
180	09-09-2024	BLOOD	71475	DSH	FEMALE	6MONTH	ANOREXIA	NON-VACCINATED	PCR	POSITIVE	LIVE
